# Freedom from atrial arrhythmia and other clinical outcomes at 5 years and beyond after catheter ablation of atrial fibrillation: a systematic review and meta-analysis

**DOI:** 10.1093/ehjqcco/qcad037

**Published:** 2023-06-19

**Authors:** Linh Ngo, Xiang Wen Lee, Mohamed Elwashahy, Pooja Arumugam, Ian A Yang, Russell Denman, Haris Haqqani, Isuru Ranasinghe

**Affiliations:** Greater Brisbane Clinical School, Medical School, The University of Queensland, Chermside, QLD 4032, Australia; Department of Cardiology, The Prince Charles Hospital, Chermside, QLD 4032, Australia; Greater Brisbane Clinical School, Medical School, The University of Queensland, Chermside, QLD 4032, Australia; Department of Cardiology, The Prince Charles Hospital, Chermside, QLD 4032, Australia; The Wollongong Hospital, Wollongong, NSW 2500, Australia; Greater Brisbane Clinical School, Medical School, The University of Queensland, Chermside, QLD 4032, Australia; Greater Brisbane Clinical School, Medical School, The University of Queensland, Chermside, QLD 4032, Australia; Department of Thoracic Medicine, The Prince Charles Hospital, Chermside, QLD 4032, Australia; Department of Cardiology, The Prince Charles Hospital, Chermside, QLD 4032, Australia; Greater Brisbane Clinical School, Medical School, The University of Queensland, Chermside, QLD 4032, Australia; Department of Cardiology, The Prince Charles Hospital, Chermside, QLD 4032, Australia; Greater Brisbane Clinical School, Medical School, The University of Queensland, Chermside, QLD 4032, Australia; Department of Cardiology, The Prince Charles Hospital, Chermside, QLD 4032, Australia

**Keywords:** Catheter ablation, Atrial fibrillation, Outcomes, Mortality, Bleeding, Stroke

## Abstract

**Aims:**

Catheter ablation of atrial fibrillation (AF) is now a mainstream procedure although long-term outcomes are uncertain. We performed a systematic review and meta-analysis of procedural outcomes at 5 years and beyond.

**Methods and results:**

We searched PubMed and Embase and after the screening, identified 73 studies (67 159 patients) reporting freedom from atrial arrhythmia, all-cause death, stroke, and major bleeding at ≥5 years after AF ablation. The pooled mean age was 59.7y, 71.5% male, 62.2% paroxysmal AF, and radiofrequency was used in 78.1% of studies. Pooled incidence of freedom from atrial arrhythmia at 5 years was 50.6% (95%CI 45.5–55.7%) after a single ablation and 69.7% [95%CI (confidence interval) 63.8–75.3%) after multiple procedures. The incidence was higher among patients with paroxysmal compared with non-paroxysmal AF after single (59.7% vs. 33.3%, *p* = 0.002) and multiple (80.8% vs. 60.6%, *p* < 0.001) ablations but was comparable between radiofrequency and cryoablation. Pooled incidences of other outcomes were 6.0% (95%CI 3.2–9.7%) for death, 2.4% (95%CI 1.4–3.7%) for stroke, and 1.2% (95%CI 0.8–2.0%) for major bleeding at 5 years. Beyond 5 years, freedom from arrhythmia recurrence remained largely stable (52.3% and 64.7% after single and multiple procedures at 10 years), while the risk of stroke and bleeding increased over time.

**Conclusion:**

Nearly 70% of patients having multiple ablations remained free from atrial arrhythmia at 5 years, with the incidence slightly decreasing beyond this period. Risk of death, stroke, and major bleeding at 5 years were low but increased over time, emphasizing the importance of long-term thromboembolism prevention and bleeding risk management.

Key learning points

- Approximately 50% of patients undergoing catheter ablation of atrial fibrillation (AF) remained free from atrial arrhythmia recurrence at 5 years after a single procedure, an incidence that increased to nearly 70% after multiple procedures.
- Incidence of AF-related adverse events among patients undergoing AF ablation was low (6.0% for mortality, 2.4% for stroke, and 1.2% for major bleeding).
- Incidence of freedom from atrial arrhythmia recurrence largely stabilized from 5 to 10-years post-ablation, while incidence of stroke and major bleeding continued to rise, emphasizing the need for long-term management of thromboembolic and bleeding risk in these patients.

## Introduction

Atrial fibrillation (AF) is the most common heart rhythm disorder encountered in clinical practice and is known to be associated with an increased risk of death and stroke or transient ischaemic attack (TIA).^1^ AF patients are also predisposed to bleeding complications given that many are on oral anticoagulant (OAC) for the prevention of thromboembolic events.^2^ Among different therapeutic options for AF, catheter ablation is shown to be superior to medications in terms of restoring normal sinus rhythm at 1–2 years post-ablation.^3–5^ Given the rapid dissemination of this procedure globally, and that patients undergoing AF ablation are often relatively young (≤65 years old) with a low profile of comorbidities,^6–8^ long-term procedural outcomes are of interest for patients and physicians. However, the risk of atrial arrhythmia recurrence and other outcomes in the years beyond the early period is poorly understood.

Most published studies report freedom from atrial arrhythmia recurrence with a prior systematic review of 6167 patients published in 2013, suggesting the risk of recurrence appear to stabilize at 5 years.^9^ However, only a few studies included in this meta-analysis reported outcomes at or beyond this period (six and four studies were included to estimate the incidence of freedom from atrial arrhythmia recurrence at 5 years after single and multiple procedures, respectively).^9^ Given AF can recur, there is a substantial desire to understand equally important clinical outcomes such as the longitudinal risk of mortality, stroke, and major bleeding in these patients yet these risks were not reported in the prior review.^9^ Other reviews, on the other hand, did not report the time-specific longitudinal risk of these adverse events, making it challenging to interpret their results.^10–13^ Moreover, the past decade has seen much more data on long-term outcomes being published, warranting an updated review of clinical outcomes of AF ablation extending to 5 years and beyond that is not limited to just atrial arrhythmia recurrence.

Accordingly, we performed a systematic review and meta-analysis of outcomes at 5 years or longer after AF ablation. We specifically examined the pooled incidence of freedom from atrial arrhythmia recurrence, all-cause mortality, stroke or TIA, and major bleeding events at 5 or more years after ablation. We also examined potential differences in outcomes among different types of AF, ablation energy used, and study design and quality.

## Methods

We followed the Preferred Reporting Items for Systematic Reviews and Meta-Analysis (PRISMA) protocol.^14^

### Search strategy

Four reviewers (LN, WL, MW, and PA) independently searched PubMed and Embase using identical search strategies (refer to Supplementary material online *Appendix Table S1* for more details) for all eligible studies published until December 2021. All studies reporting outcomes at least 5 years after AF ablation where the main ablation lesion was pulmonary vein isolation (PVI) (either segmental or circumferential) were included. After excluding duplicate records, we further excluded: (i) publications pertaining to editorial comment, review, research letter, author's reply, survey, opinion, clinical update, and study protocols; (ii) abstracts and conference proceedings to ensure reliable data extraction; (iii) studies about AF but not PVI (*including ablate and pace strategy, right atrial ablation*); (iv) studies about ablation of other arrhythmias or targets (*septal ablation*); (v) studies that did not report outcomes of interest (*cost-effectiveness, quality of life, or procedural complications*) or where data about outcomes at 5 years or more could not be extracted; (vi) studies focusing on a specific group of AF patients (*specific age group, patients with certain comorbid conditions such as heart failure, hypertrophic cardiomyopathy, valvular heart disease, common pulmonary trunk, extra-pulmonary vein triggers, congenital heart diseases, or those undergoing repeat ablation only*), or (vi) reported solely on outcomes of surgical AF ablation (*studies comparing outcomes between patients undergoing catheter ablation vs. those undergoing surgical ablation were still included*). Finally, studies that were not performed on humans, where the full text was not written in English or contained the same population reported in an included study were also excluded. LN reviewed all studies while WL, MW, and PA each reviewed approximately a third of eligible studies ensuring that all eligible studies were independently evaluated by at least two reviewers with differences resolved by consensus.

#### Quality assessment

Study quality was assessed using the tool provided by the National Institute of Health Quality which comprises of 14 questions for cohort and controlled-interventional studies and 9 questions for case-series studies.^15^ The overall quality was graded as good (when all answers were ‘No’), fair (if there were up to two ‘No’ answers), or poor (if there were at least three ‘No’ answers). Four reviewers (LN, WL, MW, and PA) independently evaluated the quality of individual studies with discrepancies resolved by consensus.

#### Data extraction and manipulation

Study data were extracted using a standardised extraction form. LN extracted all studies while WL, MW, and PA each independently extracted approximately a third of eligible studies to ensure that data extraction was performed independently by at least two reviewers with conflicts resolved by consensus. Survival data or incidence of non-fatal outcomes were extracted from the text or from Kaplan–Meier curves using the WebPlotDigitizer application.^16^ The number of events was estimated from the Kaplan–Meier curve by multiplying the number of enrolled patients with the extracted probability of experiencing the event.^17^ For studies where median and interquartile range (IQR) were reported, the mean and standard deviation (SD) were estimated using the methods outlined by Wan *et al.*^18^

#### Primary endpoints

The primary outcome was freedom from atrial arrhythmia recurrence. The definition of atrial arrhythmia recurrence varied among individual studies although most defined it as the occurrence of atrial tachyarrhythmia (AF, atrial flutter, or atrial tachycardia) lasting > 30 seconds as per treatment guidelines.^19^ When the rate of freedom from atrial arrhythmia recurrence with and without antiarrhythmic drugs was reported separately, only the result with the anti-arrhythmic drugs (AAD) was included as most studies reported outcomes with AAD only. Freedom from atrial arrhythmia recurrence was reported separately after the index procedure (single procedure) and after the last procedure (multiple procedures).

The secondary outcomes included potential clinical sequelae of AF such as all-cause mortality, stroke or TIA, and major bleeding, with the latter relating to the long-term use of anticoagulation.

#### Statistical analysis

We reported results of meta-analysis of continuous variables as the pooled mean and the corresponding 95% CI. Meta-analysis of proportions was performed with Freeman–Tukey double arcsine transformation with results reported as pooled proportion and respective 95%CI. A Random-effects model was purposefully chosen due to the differences among studies in the follow-up schedule and methods to monitor arrhythmia recurrence. The I^2^ statistic was used to evaluate the heterogeneity among studies and between-study variance τ^2^ was calculated using the restricted maximum likelihood estimator.^20^

#### Risk of bias assessment and subgroup analysis

The risk of publication bias was evaluated through visual examination of funnel plots and Egger's test was used to test for the presence of plot asymmetry.^21^ Where Egger's test indicated possible funnel plot asymmetry, a leave-one-out analysis was performed to identify the effect of leaving one study out of the meta-analysis at a time. We also performed trim and fill analysis to evaluate the change in pooled estimate when plot asymmetry was accounted for by removing studies with extreme estimates and filling in missing imputed studies based on bias-corrected estimates.^22^

Subgroup analysis was performed for different types of AF (paroxysmal vs. non-paroxysmal), two main types of ablation energy (radiofrequency ablation vs. cryoballoon), by study design (prospective vs. retrospective), and by study quality (good vs. fair vs. poor).

#### Sensitivity analysis

To evaluate whether the back transformation method would affect the pooled estimate of binary variables, we performed a sensitivity analysis by repeating the meta-analysis of the incidence of clinical outcomes with logit transformation.^23^ All analysis was performed using the ‘metafor’ package in R^24^ with a two-tailed *p* value of < 0.05 considered statistically significant.

## Results

**Figure 1:**
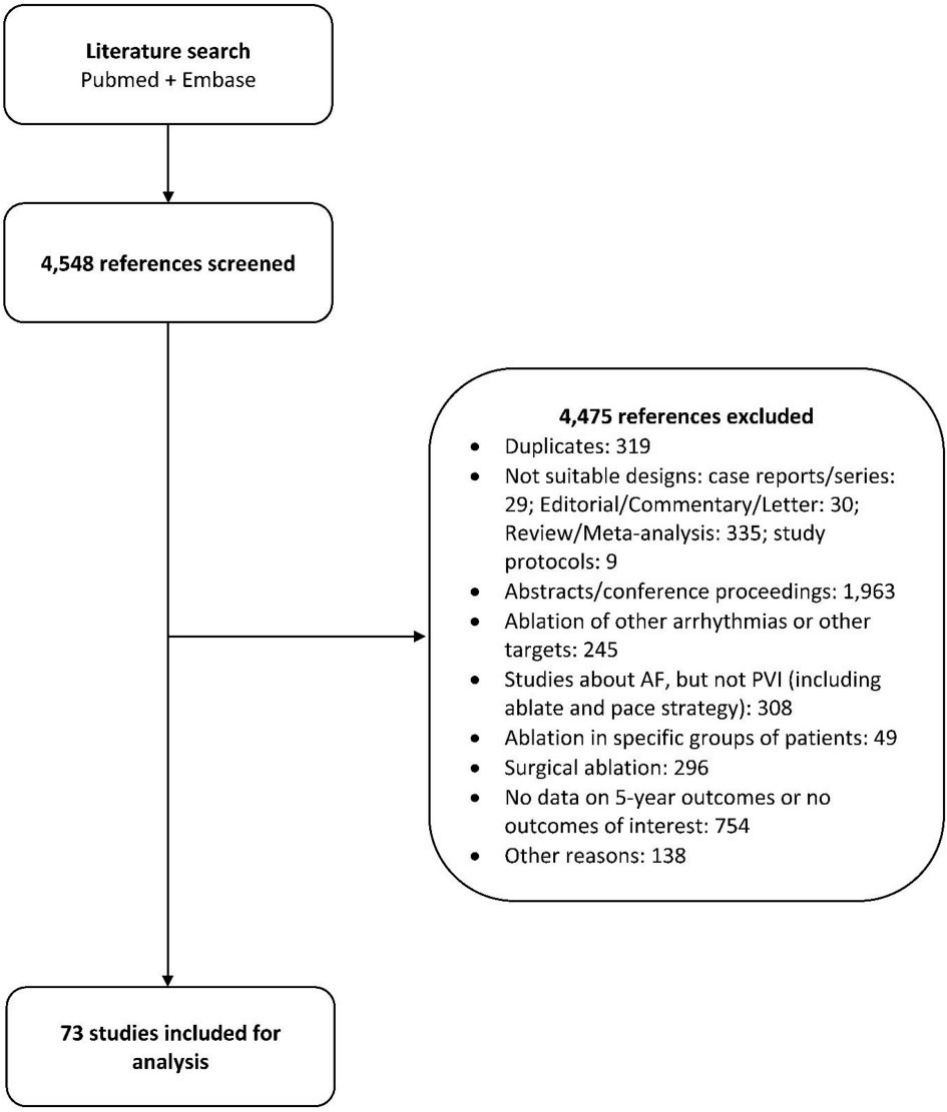
Study selection flow diagram.

We identified 4548 studies published until December 2021 that met the inclusion criteria. After applying exclusion criteria, 73 studies^25–97^ encompassing 67 159 patients were included in the systematic review and meta-analysis (*Figure 1*).

### Study characteristics

The characteristics of the included studies and patients are summarised in *Table 1*. Most (91.8%) were observational and prospective (53.4%). More than half (54.8%) had fair quality while good and poor-quality studies accounted for 34.2% and 11.0%, respectively. The pooled mean age of included patients was 59.7 years (95% CI 58.8–60.6) and 71.5% were male. Paroxysmal AF accounted for 62.2% (95%CI 51.4–72.5%) of patients. The mean AF duration before ablation was 4.2 years (95% CI 3.7–4.7). Radiofrequency was the most common energy and was used exclusively in 50 studies (68.5%). Ten studies (13.7%) used only cryoablation while seven used both radiofrequency and cryoablation (9.6%). The pooled mean follow-up time among studies was 4.8 years (4.3–5.3).

**Table 1:** Baseline characteristics of included studies

Characteristics	Number of studies (number of patients)	Summary estimate (95% CI)
Study design^a^		
Observational	67 (64 518)	91.8%
RCT	6 (2641)	8.2%
Prospective	39 (23 429)	53.4%
Retrospective	33 (42 954)	45.2%
Not reported	1 (776)	1.4%
Quality assessment^a^		
Good	25 (30 264)	34.2%
Fair	40 (33 796)	54.8%
Poor	8 (3099)	11.0%
Patient demographics		
Age (in years, mean)	71 (53 986)	59.7 (58.8–60.6)
Male (%)	73 (67 159)	71.5 (70.0–73.0)
Types of AF		
PAF	68 (43 377)	62.2 (51.4–72.5)
Non-PAF	68 (43 777)	37.8 (27.5–48.6)
AF duration (years)	49 (37 346)	4.2 (3.7–4.7)
Ablation energy		
RFA only	50 (35 576)	68.5%
CRYO only	10 (3366)	13.7%
LASER only	1 (90)	1.4%
CRYO + RFA	7 (7086)	9.6%
Not reported	5 (21 041)	6.8%
Follow-up time (years)	58 (38 302)	4.8 (4.3–5.3)
Comorbidities (% of patients)		
Hypertension	69 (51 379)	50.6 (46.9–54.3)
Previous stroke	42 (36 253)	7.8 (6.7–9.1)
Heart failure	34 (33 422)	9.6 (7.3–12.1)
Coronary artery disease	38 (30 988)	12.7 (10.2–15.4)
Cardiomyopathy	12 (12 539)	7.0 (4.6–9.8)
Vascular disease	23 (22 369)	9.1 (5.0–14.1)
Diabetes	60 (45 182)	10.7 (8.9–12.5)
Mean CHA_2_DS_2_-VASc score^b^	37 (30 806)	1.6 (1.4–1.9)
Echocardiography parameters		
LVEF (%)	50 (32 154)	59.6 (58.4–60.7)
LAD (mm)	55 (34 133)	42.6 (41.6–43.6)

Std, standard deviation; PAF, paroxysmal atrial fibrillation; RFA, radiofrequency ablation; CRYO, cryoablation; laser, laser balloon ablation; LVEF, left ventricular ejection fraction; LAD, left atrial diameter; RCT, randomized control trial. ^a^Sample size of studies with different designs and qualities were reported.

^b^CHA_2_DS_2_-VASc score is a score used to evaluate risk of experiencing thromboembolic events of AF patients in which a point each is given for the presence of congestive heart failure (C), hypertension (H), age 65–74 years old (A), diabetes (D), vascular disease (VASc) and female sex and 2 points each are given for age > = 75 years old and history of stroke (S). The total score ranges from 0 to 9 with the higher the score, the higher the risk.^98^

Many patients had comorbid hypertension (pooled proportion 50.6%, 95% CI 46.9–54.3%) while other comorbidities were less prevalent. Specifically, coronary artery disease occurred in 12.7% (95% CI 10.2–15.4%), heart failure in 9.6% (95% CI 7.3–12.1%) and diabetes in 10.7% (95% CI 8.9–12.5%). The pooled mean left ventricular ejection fraction was 59.6% (95% CI 58.4–60.7%) and the mean left atrial diameter was 42.6 mm (95% CI 41.6–43.6). The pooled mean CHA_2_DS_2_-VASc score^98^, a thromboembolic risk score, was 1.6 (95% CI 1.4–1.9). Details of individual studies included in the review are provided in Supplementary material online *Table S2* while results of study quality assessment are summarized in Supplementary material online *Table S3*.

### Primary outcome

#### Freedom from atrial arrhythmia recurrence

There was significant heterogeneity among studies regarding the definition of atrial arrhythmia recurrence, blanking period, the follow-up schedule, as well as methods to record heart rhythm during follow-up time. Most studies considered a blanking period of three months, some considered a period of 2 months, and several did not clearly define the blanking period (see Supplementary material online *Table S2* for more details).

**Figure 2:**
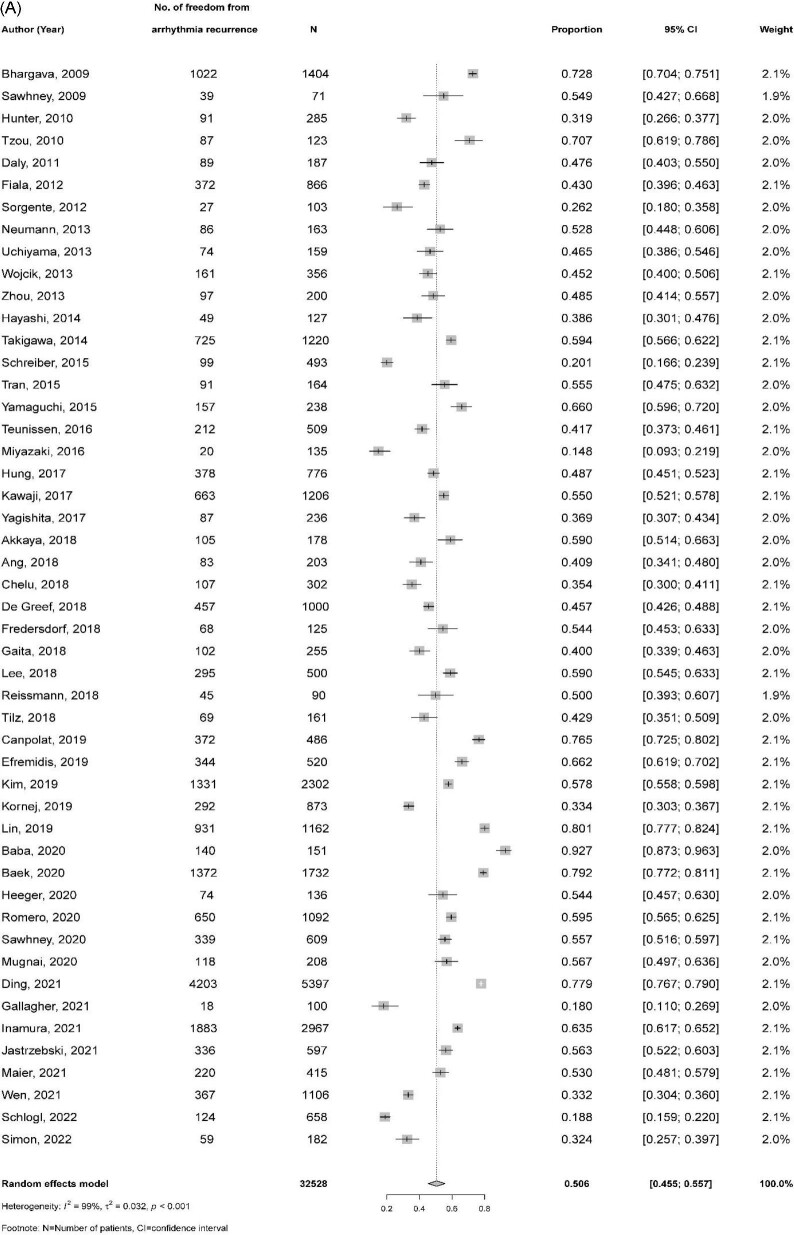

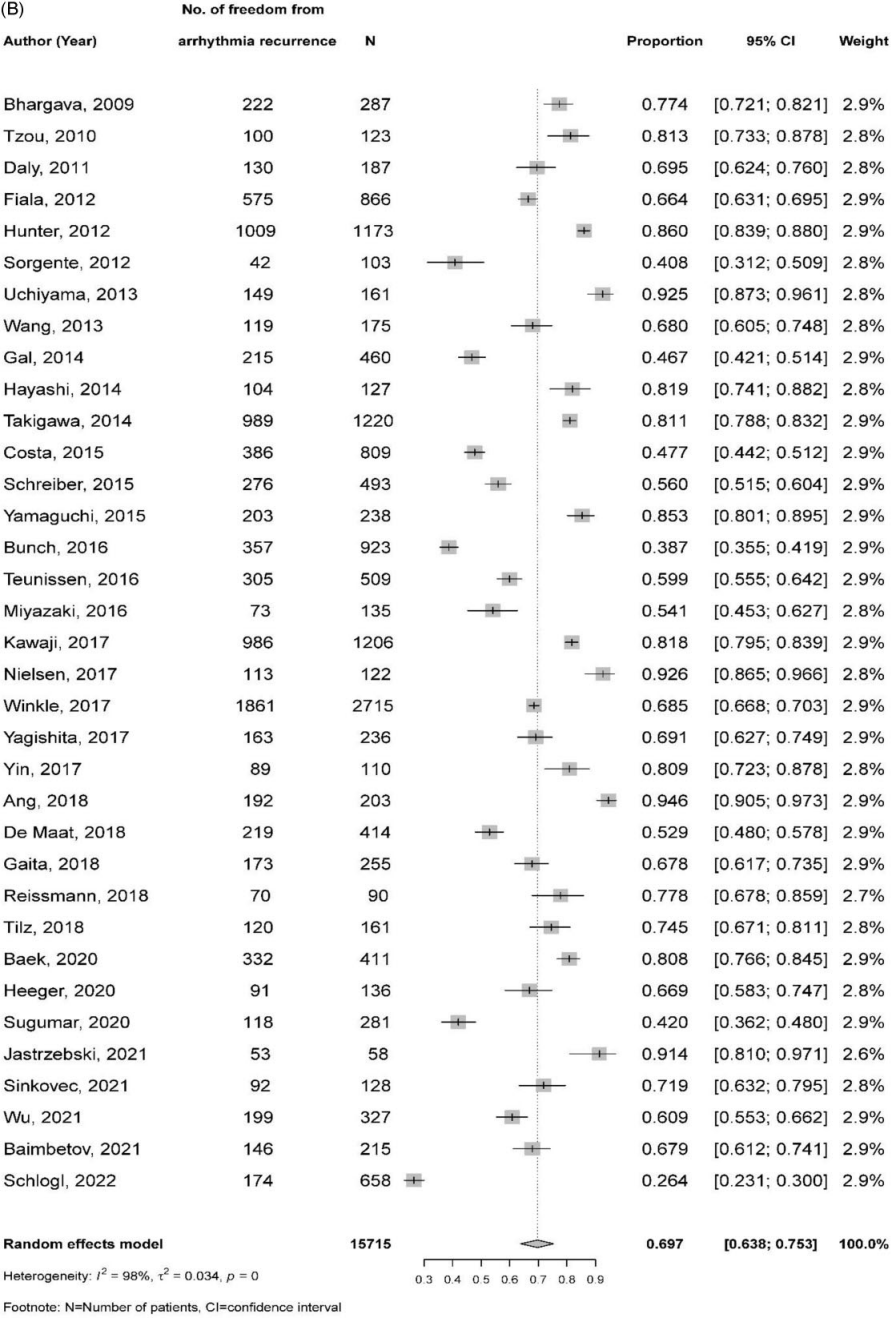
Freedom from atrial arrhythmia recurrence at 5 years following catheter ablation of atrial fibrillation: (A) after a single procedure and (B) after multiple procedures.

After a single procedure, the pooled proportion of freedom from atrial arrhythmia recurrence with AAD at 5 years post-ablation from 49 studies (32 535 patients) was 50.6% (95% CI 45.5–55.7%) with significant heterogeneity among studies (I^2 ^= 99.0%) (*Figure 2*A). In 35 studies (15 722 patients) where patients underwent multiple ablations, higher rates of freedom from atrial arrhythmia recurrence were seen (69.7%, 95%CI 63.8–75.3%) compared with a single ablation (*Figure 2*B). Freedom from atrial arrhythmia recurrence was higher among patients with paroxysmal AF after single (59.0% vs. 33.3%, *p* for subgroup difference = 0.002) and multiple ablations (80.8% vs. 60.6%, *p* < 0.001) when compared with patients with non-paroxysmal AF. Incidence of freedom from atrial arrhythmia recurrence was comparable between radiofrequency and cryoablation (49.1% vs. 56.9%, *p* = 0.077 after a single procedure and 68.0% vs. 75.7%, *p* = 0.322 after multiple procedures). The incidence of atrial arrhythmia freedom was also comparable between retrospective and prospective studies (54.1% vs. 48.7%, *p* = 0.293 after a single procedure and 71.4% vs. 69.3%, *p* = 0.719 after multiple procedures) and by study quality (the respective pooled estimates in good, fair, and poor-quality studies were 44.9%, 53.7%, and 53.3% (*p* = 0.226) after a single procedure and 71.8%, 66.6%, and 76.6% [*p* = 0.544] after multiple procedures).

### Secondary outcomes

**Figure 3:**
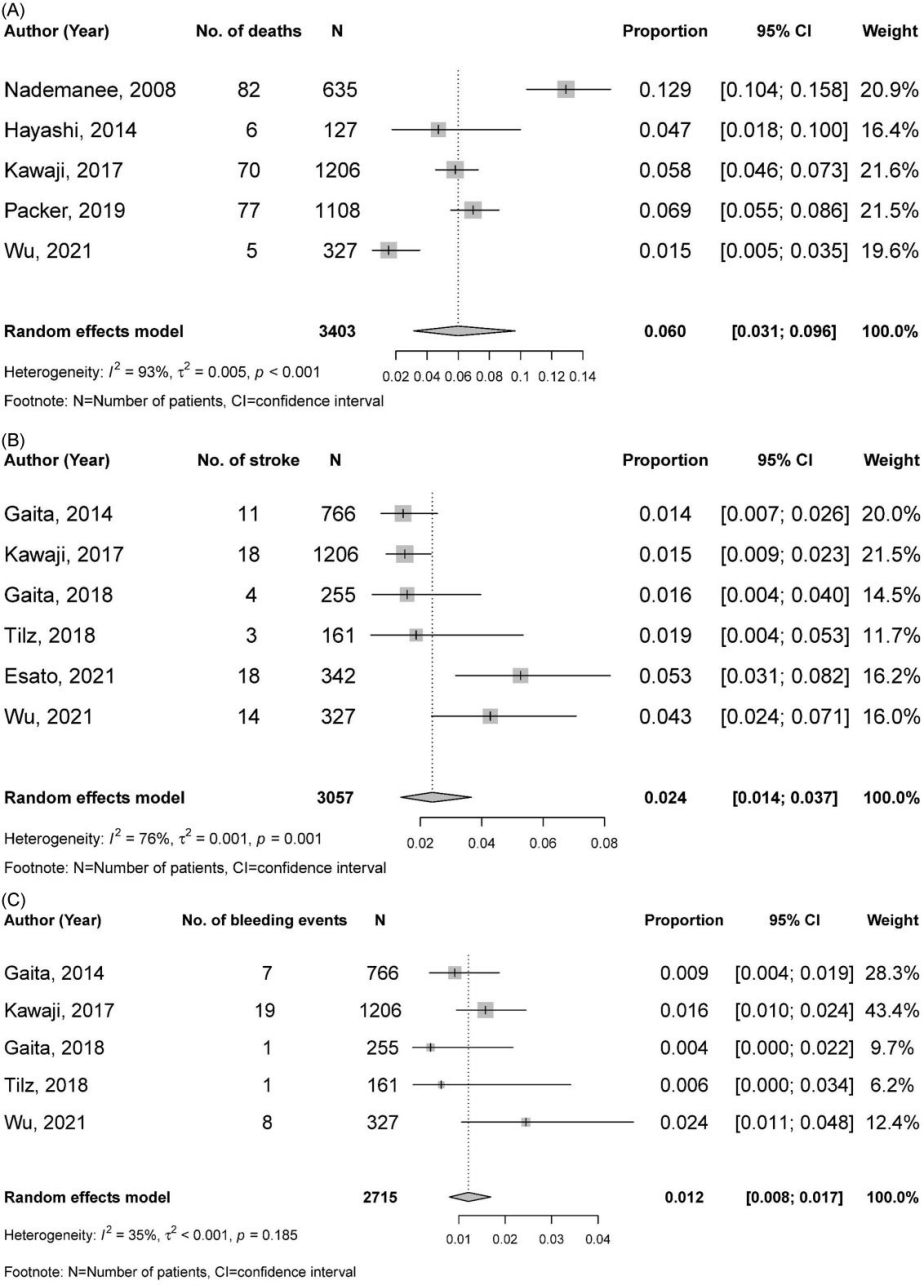
Other clinical outcomes at five years following catheter ablation of atrial fibrillation: (A) all-cause mortality; (B) stroke or transient ischaemic attack; and (C) major bleeding.

Only few studies reported on clinical outcomes other than freedom from atrial arrhythmia recurrence. Therefore, the meta-analysis of these outcomes was performed only in those undergoing multiple procedures where a sufficient sample size was available. Five studies (3403 patients)^25,41,52,76,94^ reported 5 year mortality and the pooled incidence was 6.0% (95% CI 3.1–9.6%) (*Figure 3*A). Stroke or TIA occurred less frequently with a pooled incidence of 2.4% (95%CI 1.4–3.7%, from six studies^39,52,65,69,86,94^ of 3057 patients) (*Figure 3*B). Major bleeding occurred with a pooled incidence of 1.2% (95% CI 0.7–1.7%, from five studies^39,52,65,69,94^ of 2715 patients) (*Figure 3*C). Significant heterogeneity was observed for meta-analysis of all-cause death and stroke or TIA (I^2^ of 93.0% and 76.0%, respectively), but not for major bleeding (I^2^ was 35.0%, *p* for heterogeneity = 0.185). Due to the low number of studies reporting these outcomes, subgroup analysis was not performed.

### Outcomes beyond 5 years following AF ablation

**Figure 4:**
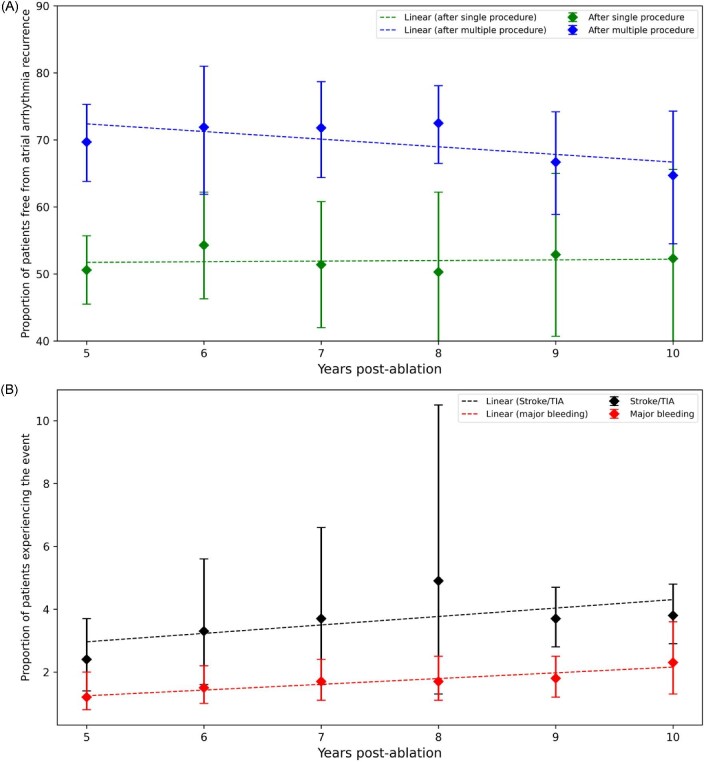
Outcomes at 5 years and beyond following catheter ablation of atrial fibrillation. (A) freedom from atrial arrhythmia recurrence and (B) incidence of stroke or transient ischaemic attack and major bleeding.

Freedom from atrial arrhythmia recurrence after a single procedure appeared to stabilise after 5 years, reaching 51.4% (95%CI 42.0–60.8%) at 7 years and 52.3% (95%CI 38.9–65.6%) at 10 years (*p* for trend = 0.060). The rate of atrial arrhythmia recurrence after multiple ablations reached 71.8% (95%CI 64.4–78.7%) at 7 years and decreased to 64.7% (95%CI 54.5–74.3%) at 10 years (*p* trend = 0.005) (*Figure 4*A). Nevertheless, only few studies were included for the meta-analysis at 10 years (six studies with 4692 patients and five studies with 2161 patients for single-procedure and multiple-procedure outcome, respectively).

When other outcomes were considered, the incidence of stroke increased from 2.4% at 5 years to 3.8% (95% CI 2.9–4.8%) at 10 years (*Figure 4*B, *p* value for trend < 0.05). Similarly, the incidence of bleeding increased over time to 2.3% (95% CI 1.3–3.6%) at 10 years following AF ablation (*p* value for trends <0.05). The pooled incidence of mortality beyond 5 years could not be estimated due to insufficient data.

### Publication bias

The funnel plots and results of Egger's test to evaluate the risk of publication bias are provided in the Supplementary material online *Appendix Figures S1–S5*. Egger's test indicated the presence of funnel plot asymmetry suggesting publication bias (*p* <0.05) for meta-analysis of freedom from atrial arrythmia after a single procedure but not after multiple procedures (*p* = 0.697). We performed a leave-one-out analysis for the outcome of freedom from atrial arrhythmia recurrence after a single procedure and found no study with a significant effect on the pooled estimate (Supplementary material online *Figure S6*). A trim-and-fill analysis showed that the pooled estimate increased to 65.0% (95%CI 58.5–71.2%) when 20 studies were imputed based on bias correction (see Supplementary material online *Figure S7* for the bias-corrected funnel plot). All *p*-values of Egger's test for the meta-analysis of the incidence of mortality, stroke or TIA, and major bleeding were >0.05, indicating a lack of funnel plot asymmetry suggesting a lack of publication bias. However, the number of studies reporting these outcomes was <10 and therefore, the ability to detect funnel plot asymmetry with Egger's test was limited.

### Sensitivity analysis

When we repeated our analysis using logit instead of Freeman–Tukey double arcsine transformation, there was no significant difference in the pooled results for freedom from atrial arrhythmia after single and multiple procedures (including subgroup analyses), as well as the pooled incidence of mortality, stroke, and bleeding (Supplementary material online *Table S4*).

## Discussion

In this systematic review, we found that about half of patients undergoing AF ablation remained free from atrial arrhythmia recurrence at 5 years after a single procedure, a success rate that increased to 7 out of 10 patients after multiple AF ablations. Beyond 5 years, freedom from atrial arrhythmia recurrence remained similar over time after a single procedure and marginally declined in patients that underwent multiple ablations, suggesting that recurrence rates largely stabilised beyond 5 years. Freedom from atrial arrhythmia recurrence was higher in patients with paroxysmal AF compared with non-paroxysmal AF, but comparable between radiofrequency and cryoablation, and among different study designs and quality. The pooled incidence of adverse outcomes such as mortality, stroke or TIA, and major bleeding was low. However, the incidence of stroke or TIA and major bleeding continued to rise beyond 5 years despite the seemingly stabilised incidence of atrial arrhythmia freedom, re-enforcing the importance of long-term thromboembolism prevention and bleeding risk management in patients that have undergone catheter ablation.

Long-term outcomes after AF ablation have been reported in a prior review published nearly 10 years ago of 19 publications including 6167 patients, although only a few studies reported outcomes extending to 5 years or more, raising uncertainty about these estimates.^9^ We extend the literature by systematically summarising procedural outcomes at 5 years or more from more than 70 studies and almost 70 000 patients, incorporating the pooled results from numerous studies published since the prior review.^9^ Our findings showed that procedural efficacy has remained the same over time, even slightly decreased after multiple ablations. This is perhaps because of the greater use of AF ablation in patients with non-paroxysmal AF, who have lower incidence of freedom from arrythmia recurrence. Another potential explanation for the static procedural efficacy is that none of advances in ablation techniques such as new ablation energy, catheter design, or ablation lesions has been unequivocally proven to have superior efficacy.^99–101^ Crucially, our analysis also provides robust data that the risk of atrial arrhythmia recurrence stabilises beyond 5 years as alluded to in the prior review,^9^ which affirms the long-term maintenance of AF ablation efficacy. This finding also suggests that those remaining free from atrial arrhythmia 5 years after the procedure could be considered as ‘cured’ from AF, which has important implications for anticoagulation therapy and for future studies investigating long-term procedural success. The continuation of anticoagulant beyond this period, however, should consider factors other than the arrhythmia status given the evidence showing patients without AF who have high CHA_2_DS_2_-VASc score are also at high risk of thromboembolic events.^102–104^ Furthermore, to confidently identify a patient as ‘free from atrial arrhythmia recurrence’ remains challenging due to the varying definition of arrhythmia recurrence, as well as different methods and frequency of rhythm monitoring. Moreover, symptomatic expression of AF varies among patients with some remaining asymptomatic, adding to the challenge of identifying atrial arrhythmia recurrence.

Although freedom from arrhythmia recurrence is frequently used to measure procedural success and efficacy, other outcomes such as all-cause mortality and stroke or TIA, are well-known clinical endpoints associated with AF.^1^ Major bleeding is another important outcome for patients undergoing AF ablation given that most still require long-term OAC for the prevention of thromboembolic events^105^ as the current recommendation for OAC continuation is based on individual patient's risk rather than rhythm status.^2^ To our knowledge, this is the first meta-analysis of these clinical endpoints at 5 years and beyond. The pooled estimates of mortality (6.0% by 5 years) and stroke/TIA (2.4% by 5 years) in our review were lower than reported for patients with AF in general enrolled in clinical trials (incidence of 3.52–3.94% *per year* for mortality and 1.27–2.2% *per year* for stroke).^106,107^ Similarly, the incidence of bleeding (1.2% by 5 years) is lower that reported in clinical trials (2.71–6.1% *per year*^106–108^) conducted in patients with AF. These observations confirm that patients undergoing AF ablation enjoy better outcomes than the general AF population even though they still face significant risk of AF-associated adverse outcomes.

These findings have several implications for clinical practice. Given freedom from atrial arrhythmia was higher after multiple ablations, clinicians should anticipate the need of multiple procedures to achieve optimal outcomes and routinely counsel patients about such possibility. If patients remain free from atrial arrhythmia at 5 years, then they are likely to remain so beyond this period, meaning that the procedure may be considered as curative in a proportion of patients. The higher success rate in paroxysmal AF also supports emerging evidence suggesting an early rhythm control strategy may lead to better long-term outcomes.^109^ Ablation energy does not appear to impact long-term efficacy although existing evidence comes mostly from studies using radiofrequency with scant data from other energy sources, highlighting a knowledge gap that needs to be addressed in future studies. Incidence of adverse outcomes such as mortality, stroke or TIA and major bleeding was low, but the risk of stroke or TIA and major bleeding continued to rise after five years despite stabilisation of the rate of freedom from atrial arrhythmia. Continuous management of thromboembolic and bleeding risk in these patients is therefore warranted. Despite these encouraging findings about AF ablation outcomes, the uptake of catheter ablation among AF patients in practice remains low^110,111^ with significant disparities such as lower rates in females^112^ and in public sector hospitals.^113^ Policies to reduce disparities and make this procedure more accessible to patients should be considered to improve AF outcomes.

This review has limitations that should be considered when interpreting the findings. We excluded all abstracts and conference proceedings, which could introduce publication bias. Indeed, Egger's test indicates the possibility of publication bias in our meta-analysis of freedom from atrial arrhythmia recurrence after a single procedure. Nevertheless, the leave-one-out analysis did not identify any study that significantly affected the pooled estimate, while the trim-and-fill analysis did not find a major change in the pooled estimate. Furthermore, such inclusion ensured that we could extract high-quality and comprehensive data for our meta-analysis. The data manipulation performed (converting median to mean and combing means of different groups) may affect the pooled estimate. However, such manipulation was necessary to maximise the number of studies included in the meta-analysis. There was significant heterogeneity among individual publications. Although we could not fully explain the high heterogeneity observed in our meta-analysis, it could be likely attributed to the significant differences among individual studies in terms of outcome definition, follow-up schedule and method, as well as results reporting. Despite these limitations, our review provides important long-term prognostic information for patients and physicians.

## Conclusion

Nearly half of the patients remained free from atrial arrhythmia at 5 years after a single AF ablation, and about 70% after multiple procedures with the risk of AF recurrence stabilising beyond this period. The incidence of other clinical outcomes such as mortality, stroke or TIA, and major bleeding was encouragingly low but increased over time, re-enforcing the importance of long-term thromboembolism prevention and bleeding risk management after catheter ablation.

## Supplementary Material

qcad037_Supplemental_File

## Data Availability

The data underlying this article are available in the article and in its online supplementary material. Any additional data will be shared on reasonable request to the corresponding author.

## References

[1] Staerk L, Sherer JA, Ko D, Benjamin EJ, Helm RH, Atrial fibrillation: epidemiology, pathophysiology, and clinical outcomes. Circ Res 2017;120:1501–1517. 28450367 10.1161/CIRCRESAHA.117.309732 PMC5500874

[2] Hindricks G, Potpara T, Dagres N, Arbelo E, Bax JJ, Blomström-Lundqvist C et al. 2020 ESC guidelines for the diagnosis and management of atrial fibrillation developed in collaboration with the European Association for Cardio-Thoracic Surgery (EACTS): the task force for the diagnosis and management of atrial fibrillation of the European Society of Cardiology (ESC) developed with the special contribution of the European Heart Rhythm Association (EHRA) of the ESC. Eur Heart J 2021;42:373–498. 32860505 10.1093/eurheartj/ehaa612

[3] Calkins H, Reynolds MR, Spector P, Sondhi M, Xu Y, Martin A et al. Treatment of atrial fibrillation with antiarrhythmic drugs or radiofrequency ablation: two systematic literature reviews and meta-analyses. Circ Arrhythm Electrophysiol 2009;2:349–361. 19808490 10.1161/CIRCEP.108.824789

[4] Mont L, Bisbal F, Hernández-Madrid A, Pérez-Castellano N, Viñolas X, Arenal A et al. Catheter ablation vs. antiarrhythmic drug treatment of persistent atrial fibrillation: a multicentre, randomized, controlled trial (SARA study). Eur Heart J 2014;35:501–507. 24135832 10.1093/eurheartj/eht457 PMC3930872

[5] Hunter RJ, Berriman TJ, Diab I, Kamdar R, Richmond L, Baker V et al. A randomized controlled trial of catheter ablation versus medical treatment of atrial fibrillation in heart failure (the CAMTAF trial). Circ Arrhythm Electrophysiol 2014;7:31–38. 24382410 10.1161/CIRCEP.113.000806

[6] Ngo L, Ali A, Ganesan A, Woodman R, Adams R, Ranasinghe I. Ten-year trends in mortality and complications following catheter ablation of atrial fibrillation. Eur Heart J Qual Care Clin Outcomes 2022;8:398–408. 34982824 10.1093/ehjqcco/qcab102

[7] Lee E, Lee So‐R, Choi E‐K, Han K‐Do, Cha M‐J, Lip GYH et al. Temporal trends of catheter ablation for patients with atrial fibrillation: a Korean nationwide population-based study. J Cardiovasc Electrophysiol 2020;31:2616–2625. 32897567 10.1111/jce.14737

[8] Kneeland PP, Fang MC. Trends in catheter ablation for atrial fibrillation in the United States. J Hosp Med 2009;4:E1–E5. 10.1002/jhm.445 PMC2919218 19753578

[9] Ganesan AN, Shipp NJ, Brooks AG, Kuklik P, Lau DH, Lim HS et al. Long-term outcomes of catheter ablation of atrial fibrillation: a systematic review and meta-analysis. J Am Heart Assoc 2013;2:e004549. 23537812 10.1161/JAHA.112.004549 PMC3647286

[10] Barra S, Baran J, Narayanan K, Boveda S, Fynn S, Heck P et al. Association of catheter ablation for atrial fibrillation with mortality and stroke: a systematic review and meta-analysis. Int J Cardiol 2018;266:136–142. 29887429 10.1016/j.ijcard.2018.03.068

[11] Asad ZUlA, Yousif A, Khan MS, Al-Khatib SM, Stavrakis S. Catheter ablation versus medical therapy for atrial fibrillation: a systematic review and meta-analysis of randomized controlled trials. Circ Arrhythm Electrophysiol 2019;12:e007414. 31431051 10.1161/CIRCEP.119.007414

[12] Deshpande R, Alkhadra Y, Singanallur P, Botchway A, Labedi M. Outcomes of catheter ablation versus antiarrhythmic therapy in patients with atrial fibrillation: a systematic review and meta-analysis. J Interv Card Electrophysiol 2022. 10.1007/s10840-022-01365-z 36057733

[13] Maduray K, Moneruzzaman Md, Changwe GJ, Zhong J. Benefits and risks associated with long-term oral anticoagulation after successful atrial fibrillation catheter ablation: systematic review and meta-analysis. Clin Appl Thromb Hemost 2022;28:10760296221118480. 35924410 10.1177/10760296221118480 PMC9358599

[14] Moher D . Preferred reporting items for systematic reviews and meta-analyses: the PRISMA statement. Ann Intern Med 2009;151:264–269, W64. 19622511 10.7326/0003-4819-151-4-200908180-00135

[15] National Heart, Lung and Blood Institute . Study quality assessment tool. 2014 Available at: https://www.nhlbi.nih.gov/health-topics/study-quality-assessment-tools.

[16] Rohatgi A . Webplotdigitizer: version 4.5. 2021 Available at: https://automeris.io/WebPlotDigitizer.

[17] Duchateau L, Collette L, Sylvester R, Pignon J P. Estimating number of events from the Kaplan-Meier curve for incorporation in a literature-based meta-analysis: what you don't see you can't get! Biometrics 2000;56:886–892. 10985232 10.1111/j.0006-341x.2000.00886.x

[18] Wan X, Wang W, Liu J, Tong T. Estimating the sample mean and standard deviation from the sample size, median, range and/or interquartile range. BMC Med Res Methodol 2014;14:135. 25524443 10.1186/1471-2288-14-135 PMC4383202

[19] Calkins H, Hindricks G, Cappato R, Kim Y-H, Saad EB, Aguinaga L et al. 2017 HRS/EHRA/ECAS/APHRS/SOLAECE expert consensus statement on catheter and surgical ablation of atrial fibrillation: executive summary. J Arrhythm 2017;33:369–409. 29021841 10.1016/j.joa.2017.08.001 PMC5634725

[20] Veroniki AA, Jackson D, Viechtbauer W, Bender R, Bowden J, Knapp G et al. Methods to estimate the between-study variance and its uncertainty in meta-analysis. Res Synth Methods 2016;7:55–79. 26332144 10.1002/jrsm.1164 PMC4950030

[21] Egger M, Smith GD, Schneider M, Minder C. Bias in meta-analysis detected by a simple, graphical test. BMJ 1997;315:629–634. 9310563 10.1136/bmj.315.7109.629 PMC2127453

[22] Shi L, Lin L. The trim-and-fill method for publication bias: practical guidelines and recommendations based on a large database of meta-analyses. Medicine (Baltimore) 2019;98:e15987. 31169736 10.1097/MD.0000000000015987 PMC6571372

[23] Schwarzer G, Chemaitelly H, Abu‐Raddad LJ, Rücker G. Seriously misleading results using inverse of Freeman-Tukey double arcsine transformation in meta-analysis of single proportions. Res Synth Methods 2019;10:476–483. 30945438 10.1002/jrsm.1348 PMC6767151

[24] Viechtbauer W . Conducting Meta-Analyses in R with the metafor Package. J Stat Softw 2010;36:1–48.

[25] Nademanee K, Schwab MC, Kosar EM, Karwecki M, Moran MD, Visessook N et al. Clinical outcomes of catheter substrate ablation for high-risk patients with atrial fibrillation. J Am Coll Cardiol 2008;51:843–849. 18294570 10.1016/j.jacc.2007.10.044

[26] Bhargava M, Di Biase L, Mohanty P, Prasad S, Martin DO, Williams-Andrews M et al. Impact of type of atrial fibrillation and repeat catheter ablation on long-term freedom from atrial fibrillation: results from a multicenter study. Heart Rhythm 2009;6:1403–1412. 19716348 10.1016/j.hrthm.2009.06.014

[27] Sawhney N, Anousheh R, Chen W-C, Narayan S, Feld GK. Five-year outcomes after segmental pulmonary vein isolation for paroxysmal atrial fibrillation. Am J Cardiol 2009;104:366–372. 19616669 10.1016/j.amjcard.2009.03.044 PMC2892619

[28] Hunter RJ, Berriman TJ, Diab I, Baker V, Finlay M, Richmond L et al. Long-term efficacy of catheter ablation for atrial fibrillation: impact of additional targeting of fractionated electrograms. Heart 2010;96:1372–1378. 20483892 10.1136/hrt.2009.188128

[29] Tzou WS, Marchlinski FE, Zado ES, Lin D, Dixit S, Callans DJ et al. Long-term outcome after successful catheter ablation of atrial fibrillation. Circ Arrhythm Electrophysiol 2010;3:237–242. 20335557 10.1161/CIRCEP.109.923771

[30] Daly M, Melton I, Crozier I. Pulmonary vein ablation for atrial fibrillation: the Christchurch, New Zealand experience. N Z Med J 2011;124:39–47. 21964012

[31] Fialaa M, Škňouřila L, Tomanb O, Pindora J, Bulkováa V, Chovančíka J et al. Long-term results of catheter ablation for atrial fibrillation in 866 patients. Cor Vasa 2012;54:e361–e368.

[32] Hunter RJ, Mccready J, Diab I, Page SP, Finlay M, Richmond L et al. Maintenance of sinus rhythm with an ablation strategy in patients with atrial fibrillation is associated with a lower risk of stroke and death. Heart 2012;98:48–53. 21930724 10.1136/heartjnl-2011-300720

[33] Sorgente A, Tung P, Wylie J, Josephson ME. Six year follow-up after catheter ablation of atrial fibrillation: a palliation more than a true cure. Am J Cardiol 2012;109:1179–1186. 22245414 10.1016/j.amjcard.2011.11.058

[34] Neumann T, Wojcik M, Berkowitsch A, Erkapic D, Zaltsberg S, Greiss H et al. Cryoballoon ablation of paroxysmal atrial fibrillation: 5-year outcome after single procedure and predictors of success. Europace 2013;15:1143–1149. 10.1093/europace/eut021 23419659

[35] Uchiyama T, Miyazaki S, Taniguchi H, Komatsu Y, Kusa S, Nakamura H et al. Six-year follow-up of catheter ablation in paroxysmal atrial fibrillation. Circ J 2013;77:2722–2727. 23924888 10.1253/circj.cj-13-0468

[36] Wang K, Chang D, Chu Z, Yang Y, Gao L, Zhang S et al. Denervation as a common mechanism underlying different pulmonary vein isolation strategies for paroxysmal atrial fibrillation: evidenced by heart rate variability after ablation. ScientificWorldJournal 2013;2013:569564. 24058286 10.1155/2013/569564 PMC3766572

[37] Wójcik M, Erkapic D, Berkowitsch A, Zaltsberg S, Greiss H, Schmitt J et al. Ipsilateral circumferential radiofrequency ablation of atrial fibrillation with irrigated tip catheter: long-term outcome and pre-procedural predictors. Circ J 2013;77:2280–2287. 23811678 10.1253/circj.cj-13-0275

[38] Zhou G, Chen S, Chen G, Zhang F, Meng W, Yan Y et al. Procedural arrhythmia termination and long-term single-procedure clinical outcome in patients with non-paroxysmal atrial fibrillation. J Cardiovasc Electrophysiol 2013;24:1092–1100. 23790106 10.1111/jce.12193

[39] Gaita F, Sardi D, Battaglia A, Gallo C, Toso E, Michielon A et al. Incidence of cerebral thromboembolic events during long-term follow-up in patients treated with transcatheter ablation for atrial fibrillation. Europace 2014;16:980–986. 24446510 10.1093/europace/eut406

[40] Gal P, Aarntzen AESM, Smit JJJ, Adiyaman A, Misier ARR, Delnoy PPHM et al. Conventional radiofrequency catheter ablation compared to multi-electrode ablation for atrial fibrillation. Int J Cardiol 2014;176:891–895. 25156854 10.1016/j.ijcard.2014.08.034

[41] Hayashi M, Kaneko S, Shimano M, Ohashi T, Kubota R, Takeshita K et al. Efficacy and safety of radiofrequency catheter ablation for atrial fibrillation in chronic hemodialysis patients. Nephrol Dial Transplant 2014;29:160–167. 24166462 10.1093/ndt/gft233

[42] Takigawa M, Takahashi A, Kuwahara T, Okubo K, Takahashi Y, Watari Y et al. Long-term follow-up after catheter ablation of paroxysmal atrial fibrillation: the incidence of recurrence and progression of atrial fibrillation. Circ Arrhythm Electrophysiol 2014;7:267–273. 24610740 10.1161/CIRCEP.113.000471

[43] Costa FM, Ferreira AM, Oliveira S, Santos PG, Durazzo A, Carmo P et al. Left atrial volume is more important than the type of atrial fibrillation in predicting the long-term success of catheter ablation. Int J Cardiol 2015;184:56–61. 25697871 10.1016/j.ijcard.2015.01.060

[44] Karasoy D, Gislason GH, Hansen J, Johannessen A, Køber L, Hvidtfeldt M et al. Oral anticoagulation therapy after radiofrequency ablation of atrial fibrillation and the risk of thromboembolism and serious bleeding: long-term follow-up in nationwide cohort of Denmark. Eur Heart J 2015;36:307–14a. 25368205 10.1093/eurheartj/ehu421

[45] Noseworthy PA, Kapa S, Deshmukh AJ, Madhavan M, Van Houten H, Haas LR et al. Risk of stroke after catheter ablation versus cardioversion for atrial fibrillation: a propensity-matched study of 24,244 patients. Heart Rhythm 2015;12:1154–1161. 25708883 10.1016/j.hrthm.2015.02.020

[46] Schreiber D, Rostock T, Fröhlich M, Sultan A, Servatius H, Hoffmann BA et al. Five-year follow-up after catheter ablation of persistent atrial fibrillation using the stepwise approach and prognostic factors for success. Circ Arrhythm Electrophysiol 2015;8:308–317. 25744570 10.1161/CIRCEP.114.001672

[47] Tran VN, Tessitore E, Gentil-Baron P, Jannot A-S, Sunthorn H, Burri H et al. Thromboembolic events 7-11 years after catheter ablation of atrial fibrillation. Pacing Clin. Electrophysiol. 2015;38:499–506. 25626468 10.1111/pace.12588

[48] Yamaguchi Y, Sohara H, Takeda H, Nakamura Y, Ihara M, Higuchi S et al. Long-term results of radiofrequency hot balloon ablation in patients with paroxysmal atrial fibrillation: safety and rhythm outcomes. J Cardiovasc Electrophysiol 2015;26:1298–1306. 26331460 10.1111/jce.12820

[49] Bunch TJ, May HT, Bair TL, Jacobs V, Crandall BG, Cutler M et al. The impact of age on 5-year outcomes after atrial fibrillation catheter ablation. J Cardiovasc Electrophysiol 2016;27:141–146. 26443666 10.1111/jce.12849

[50] Teunissen C, Kassenberg W, Van Der Heijden JF, Hassink RJ, Van Driel VJHM, Zuithoff NPA et al. Five-year efficacy of pulmonary vein antrum isolation as a primary ablation strategy for atrial fibrillation: a single-centre cohort study. Europace 2016;18:1335–1342. 26838694 10.1093/europace/euv439

[51] Hung Y, Lo Li-W, Lin Y-J, Chang S-L, Hu Yu-F, Chung Fa-Po et al. Characteristics and long-term catheter ablation outcome in long-standing persistent atrial fibrillation patients with non-pulmonary vein triggers. Int J Cardiol 2017;241:205–211. 28456483 10.1016/j.ijcard.2017.04.050

[52] Kawaji T, Shizuta S, Morimoto T, Aizawa T, Yamagami S, Yoshizawa T et al. Very long-term clinical outcomes after radiofrequency catheter ablation for atrial fibrillation: a large single-center experience. Int J Cardiol 2017;249:204–213. 28964553 10.1016/j.ijcard.2017.09.023

[53] Miyazaki S, Taniguchi H, Kusa S, Nakamura H, Hachiya H, Hirao K et al. Five-year follow-up outcome after catheter ablation of persistent atrial fibrillation using a sequential biatrial linear defragmentation approach: what does atrial fibrillation termination during the procedure imply? Heart Rhythm, 2017;14:34–40. 10.1016/j.hrthm.2016.08.041 27590435

[54] Nielsen JC, Johannessen A, Raatikainen P, Hindricks G, Walfridsson H, Pehrson SM et al. Long-term efficacy of catheter ablation as first-line therapy for paroxysmal atrial fibrillation: 5-year outcome in a randomised clinical trial. Heart, 2017;103:368–376. 27566295 10.1136/heartjnl-2016-309781

[55] Saliba W, Schliamser JE, Lavi I, Barnett-Griness O, Gronich N, Rennert G. Catheter ablation of atrial fibrillation is associated with reduced risk of stroke and mortality: a propensity score—matched analysis. Heart Rhythm 2017;14:635–642. 28189823 10.1016/j.hrthm.2017.02.001

[56] Winkle RA, Mead RH, Engel G, Kong MH, Fleming W, Salcedo J et al. Impact of obesity on atrial fibrillation ablation: patient characteristics, long-term outcomes, and complications. Heart Rhythm 2017;14:819–827. 28232261 10.1016/j.hrthm.2017.02.023

[57] Yagishita A, Yamauchi Y, Sato H, Yamashita S, Hirao T, Miyamoto T et al. Efficacy of catheter ablation and concomitant antiarrhythmic drugs on the reduction of the arrhythmia burden in patients with long-standing persistent atrial fibrillation. J Atr Fibrillation 2017;10:1649. 29250243 10.4022/jafib.1649 PMC5725751

[58] Yin X, Zhao Z, Gao L, Chang D, Xiao X, Zhang R et al. Frequency gradient within coronary sinus predicts the long-term outcome of persistent atrial fibrillation catheter ablation. J Am Heart Assoc 2017;6:e004869. 10.1161/JAHA.116.004869 PMC5524018 28255079

[59] Akkaya E, Berkowitsch A, Zaltsberg S, Greiss H, Hamm CW, Sperzel J et al. Five-year outcome and predictors of success after second-generation cryoballoon ablation for treatment of symptomatic atrial fibrillation. Int J Cardiol 2018;266:106–111. 29887425 10.1016/j.ijcard.2018.03.069

[60] Ang R, Hunter RJ, Lim WY, Opel A, Ullah W, Providencia R et al.. Long term outcome and pulmonary vein reconnection of patients undergoing cryoablation and/or radiofrequency ablation: results from the cryo versus RF Trial. J Atr Fibrillation 2018;11:2072. 10.4022/jafib.2072 PMC6533809 31139275

[61] Chelu MG, King JB, Kholmovski EG, Ma J, Gal P, Marashly Q et al. Atrial fibrosis by late gadolinium enhancement magnetic resonance imaging and catheter ablation of atrial fibrillation: 5-year follow-up data. J Am Heart Assoc 2018;7:e006313. 30511895 10.1161/JAHA.117.006313 PMC6405558

[62] De Greef Y, Schwagten B, Chierchia GB, De Asmundis C, Stockman D, Buysschaert I. Diagnosis-to-ablation time as a predictor of success: early choice for pulmonary vein isolation and long-term outcome in atrial fibrillation: results from the Middelheim-PVI Registry. Europace 2018;20:589–595. 28340103 10.1093/europace/euw426

[63] De Maat GE, Mulder B, Berretty WL, Al-Jazairi MIH, Tan YES, Wiesfeld ACP et al. Obesity is associated with impaired long-term success of pulmonary vein isolation: a plea for risk factor management before ablation. Open Heart 2018;5:e000771. 29862033 10.1136/openhrt-2017-000771 PMC5976117

[64] Fredersdorf S, Fenzl C, Jungbauer C, Weber S, Von Bary C, Dietl A et al. Long-term outcomes and predictors of recurrence after pulmonary vein isolation with multielectrode ablation catheter in patients with atrial fibrillation. J Cardiovasc Med (Hagerstown) 2018;19:148–154. 29432401 10.2459/JCM.0000000000000631

[65] Gaita F, Scaglione M, Battaglia A, Matta M, Gallo C, Galatà M et al. Very long-term outcome following transcatheter ablation of atrial fibrillation. Are results maintained after 10 years of follow up? Europace 2018;20:443–450. 28340043 10.1093/europace/eux008

[66] Kim D-H, Lee D-In, Ahn J, Lee K-No, Roh S-Y, Shim J et al. Ischemic stroke risk during long-term follow up in patients with successful catheter ablation for atrial fibrillation in Korea. PLoS One 2018;13:e0201061. 30024976 10.1371/journal.pone.0201061 PMC6053230

[67] Lee K-No, Roh S-Y, Baek Y-S, Park H-S, Ahn J, Kim D-H et al. Long-term clinical comparison of procedural end points after pulmonary vein isolation in paroxysmal atrial fibrillation: elimination of nonpulmonary vein triggers versus noninducibility. Circ Arrhythm Electrophysiol 2018;11:e005019. 29431632 10.1161/CIRCEP.117.005019

[68] Reissmann B, Budelmann T, Wissner E, Schlüter M, Heeger C-H, Mathew S et al. Five-year clinical outcomes of visually guided laser balloon pulmonary vein isolation for the treatment of paroxysmal atrial fibrillation. Clin Res Cardiol 2018;107:405–412. 29285621 10.1007/s00392-017-1199-6

[69] Tilz RR, Heeger C-H, Wick A, Saguner AM, Metzner A, Rillig A et al. Ten-year clinical outcome after circumferential pulmonary vein isolation utilizing the Hamburg approach in patients with symptomatic drug-refractory paroxysmal atrial fibrillation. Circ Arrhythm Electrophysiol 2018;11:e005250. 29449353 10.1161/CIRCEP.117.005250

[70] Canpolat U, Kocyigit D, Yalcin MU, Coteli C, Sener YZ, Oksul M et al. Long-term outcomes of pulmonary vein isolation using second-generation cryoballoon during atrial fibrillation ablation. Pacing Clin Electrophysiol 2019;42:910–921. 31106431 10.1111/pace.13721

[71] Efremidis M, Letsas KP, Georgopoulos S, Karamichalakis N, Vlachos K, Lioni L et al. Safety, long-term outcomes and predictors of recurrence following a single catheter ablation procedure for atrial fibrillation. Acta Cardiol 2019;74:319–324. 30303043 10.1080/00015385.2018.1494114

[72] Gedikli Ö, Mohanty S, Trivedi C, Gianni C, Chen Q, Della Rocca DG et al. Impact of dense “smoke” detected on transesophageal echocardiography on stroke risk in patients with atrial fibrillation undergoing catheter ablation. Heart Rhythm 2019;16:351–357. 30312757 10.1016/j.hrthm.2018.10.004

[73] Kim JOk, Shim J, Lee S-H, Yu HT, Kim T-H, Uhm J-S et al. Clinical characteristics and rhythm outcome of catheter ablation of hemodynamically corrected valvular atrial fibrillation. J Cardiol 2019;73:488–496. 30850308 10.1016/j.jjcc.2018.10.014

[74] Kornej J, Schumacher K, Zeynalova S, Sommer P, Arya A, Weiß M et al. Time-dependent prediction of arrhythmia recurrences during long-term follow-up in patients undergoing catheter ablation of atrial fibrillation. Leipzig Heart Center AF Ablation Registry Sci Rep 2019;9:7112. 10.1038/s41598-019-43644-2 PMC6506496 31068651

[75] Lin Y, Wu H-K, Wang Te-H, Chen T-H, Lin Yu-S. Trend and risk factors of recurrence and complications after arrhythmias radiofrequency catheter ablation: a nation-wide observational study in Taiwan. BMJ Open 2019;9:e023487. 10.1136/bmjopen-2018-023487 PMC6549656 31152025

[76] Packer DL, Mark DB, Robb RA, Monahan KH, Bahnson TD, Poole JE et al. Effect of catheter ablation vs antiarrhythmic drug therapy on mortality, stroke, bleeding, and cardiac arrest among patients with atrial fibrillation: the CABANA randomized clinical trial. JAMA 2019;321:1261–1274. 30874766 10.1001/jama.2019.0693 PMC6450284

[77] Baba M, Yoshida K, Naruse Y, Hattori A, Yui Y, Kimata A et al. Predictors of recurrence after catheter ablation of paroxysmal atrial fibrillation in different follow-up periods. Medicina (Kaunas) 2020;56:465. 32932837 10.3390/medicina56090465 PMC7557836

[78] Baek Y-S, Choi J-Il, Kim YGi, Lee K-No, Roh S-Y, Ahn J et al. Atrial substrate underlies the recurrence after catheter ablation in patients with atrial fibrillation. J Clin Med 2020;9:1–13. 10.3390/jcm9103164 PMC7601892 33007810

[79] Heeger C-H, Subin B, Wissner E, Fink T, Mathew S, Maurer T et al. Second-generation cryoballoon-based pulmonary vein isolation: lessons from a five-year follow-up. Int J Cardiol 2020;312:73–80. 32241572 10.1016/j.ijcard.2020.03.062

[80] Kis Z, Martirosyan M, Hendriks AA, Theuns D, Bhagwandien R, Wijchers S et al. High cerebrovascular thromboembolic event rate long after unsuccessful catheter ablation for atrial fibrillation. J Atr Fibrillation 2020;13:2294. 34950301 10.4022/jafib.2294 PMC8691342

[81] Kriatselis C, Unruh T, Kaufmann J, Gerds-Li J-H, Kelle S, Gebker R et al. Long-term left atrial remodeling after ablation of persistent atrial fibrillation: 7-year follow-up by cardiovascular magnetic resonance imaging. J Interv Card Electrophysiol 2020;58:21–27. 31230178 10.1007/s10840-019-00584-1

[82] Romero J, Di Biase L, Mohanty S, Trivedi C, Patel K, Parides M et al. Long-term outcomes of left atrial appendage electrical isolation in patients with nonparoxysmal atrial fibrillation: a propensity score-matched analysis. Circ Arrhythm Electrophysiol 2020;13:e008390. 32998529 10.1161/CIRCEP.120.008390

[83] Sawhney V, Schilling RJ, Providencia R, Cadd M, Perera D, Chatha S et al. Cryoablation for persistent and longstanding persistent atrial fibrillation: results from a multicentre European registry. Europace 2020;22:375–381. 31808520 10.1093/europace/euz313

[84] Sugumar H, Nanayakkara S, Chieng D, Wong GR, Parameswaran R, Anderson RD et al. Arrhythmia recurrence is more common in females undergoing multiple catheter ablation procedures for persistent atrial fibrillation: time to close the gender gap. Heart Rhythm 2020;17:692–698. 31866381 10.1016/j.hrthm.2019.12.013

[85] Ding WY, Yang P‐S, Jang E, Gupta D, Sung J‐H, Joung B et al. Impact of abdominal obesity on outcomes of catheter ablation in Korean patients with atrial fibrillation. Int J Clin Pract 2021;75:e14696. 34338415 10.1111/ijcp.14696 PMC11475307

[86] Esato M, An Y, Ogawa H, Wada H, Hasegawa K, Tsuji H et al. Major adverse cardiovascular events and mortality after catheter ablation in Japanese patients with atrial fibrillation: the Fushimi AF Registry. Heart Vessels 2021;36:1219–1227. 33575844 10.1007/s00380-021-01796-0

[87] Gallagher MM, Yi G, Gonna H, Leung LWM, Harding I, Evranos B et al. Multi-catheter cryotherapy compared with radiofrequency ablation in long-standing persistent atrial fibrillation: a randomized clinical trial. Europace 2021;23:370–379. 33188692 10.1093/europace/euaa289

[88] Inamura Y, Nitta J, Inaba O, Sato A, Takamiya T, Murata K et al. Presence of non-pulmonary vein foci in patients with atrial fibrillation undergoing standard ablation of pulmonary vein isolation: clinical characteristics and long-term ablation outcome. Int J Cardiol Heart Vasc 2021;32:100717. 33532545 10.1016/j.ijcha.2021.100717 PMC7822950

[89] Jastrzębski M, Kiełbasa G, Fijorek K, Bednarski A, Kusiak A, Sondej T et al. Outcomes of atrial fibrillation ablation program based on single-shot techniques. Postepy Kardiol Interwencyjnej 2021;16:466–473. 10.5114/aic.2020.101773 PMC7863826 33598021

[90] Maier J, Blessberger H, Nahler A, Hrncic D, Fellner A, Reiter C et al. Cardiac computed tomography-derived left atrial volume index as a predictor of long-term success of cryo-ablation in patients with atrial fibrillation. Am J Cardiol 2021;140:69–77. 33152317 10.1016/j.amjcard.2020.10.061

[91] Mugnai G, Paparella G, Overeinder I, Ströker E, Sieira J, Bisignani A et al. Long-term clinical outcomes after single freeze cryoballoon ablation for paroxysmal atrial fibrillation: a 5-year follow-up. J. Interv Card Electrophysiol. 2021;61:87–93. 32472280 10.1007/s10840-020-00788-w

[92] Šinkovec Mž, Jan Mž, Antolič B, Klemen L, Pernat A. Long-term outcomes after catheter ablation of atrial fibrillation: single centre experience. Slov. Med. J 2021;90:21–37.

[93] Wen S, Pislaru C, Monahan KH, Barnes SM, Hodge DO, Packer DL et al. Arrhythmia recurrence after atrial fibrillation ablation: impact of warfarin vs. non-vitamin K antagonist oral anticoagulants. Cardiovasc Drugs Ther 2022;36:891–901. 34003404 10.1007/s10557-021-07200-3

[94] Wu G, Huang He, Cai L, Yang Y, Liu Xu, Yu Bo et al. Long-term observation of catheter ablation vs. pharmacotherapy in the management of persistent and long-standing persistent atrial fibrillation (CAPA study). Europace 2021;23:731–739. 33367669 10.1093/europace/euaa356

[95] Baimbetov AK, Abzaliev KB, Jukenova AM, Bizhanov KA, Bairamov BA, Ualiyeva AYe. The efficacy and safety of cryoballoon catheter ablation in patients with paroxysmal atrial fibrillation. Ir J Med Sci 2022;191:187–193. 10.1007/s11845-021-02560-z 33638796

[96] Schlögl S, Schlögl KS, Haarmann H, Bengel P, Bergau L, Rasenack E et al. Remote magnetic navigation versus manual catheter ablation of atrial fibrillation: a single center long-term comparison. Pacing Clin Electrophysiol 2022;45:14–22. 34687054 10.1111/pace.14392

[97] Simon J, El Mahdiui M, Smit JM, Száraz L, Rosendael AR, Herczeg S et al. Left atrial appendage size is a marker of atrial fibrillation recurrence after radiofrequency catheter ablation in patients with persistent atrial fibrillation. Clin Cardiol 2022;45:273–281. 34799870 10.1002/clc.23748 PMC8922535

[98] Lip GYH, Nieuwlaat R, Pisters R, Lane DA, Crijns HJGM. Refining clinical risk stratification for predicting stroke and thromboembolism in atrial fibrillation using a novel risk factor-based approach: the euro heart survey on atrial fibrillation. Chest 2010;137:263–272. 19762550 10.1378/chest.09-1584

[99] Chen Yi-He, Lu Z-Y, Xiang Y-, Hou J-W, Wang Q, Lin H et al. Cryoablation vs. radiofrequency ablation for treatment of paroxysmal atrial fibrillation: a systematic review and meta-analysis. Europace 2017;19:784–794. 28065886 10.1093/europace/euw330

[100] Virk SA, Ariyaratnam J, Bennett RG, Kumar S. Updated systematic review and meta-analysis of the impact of contact force sensing on the safety and efficacy of atrial fibrillation ablation: discrepancy between observational studies and randomized control trial data. Europace 2019;21:239–249. 30544134 10.1093/europace/euy266

[101] Chen Yi-He, Lin H, Wang Q, Hou J-W, Li Yi-G. Efficacy and safety of adjunctive substrate modification during pulmonary vein isolation for atrial fibrillation: a meta-analysis. Heart Lung Circ 2020;29:422–436. 31072768 10.1016/j.hlc.2019.01.018

[102] Parsons C, Patel SI, Cha S, Shen W-K, Desai S, Chamberlain AM et al. CHA(_2_)DS(_2_)-VASc score: a predictor of thromboembolic events and mortality in patients with an implantable monitoring device without atrial fibrillation. Mayo Clin Proc 2017;92:360–369. 28259228 10.1016/j.mayocp.2016.10.008 PMC5641434

[103] Koene RJ, Alraies MC, Norby FL, Soliman EZ, Maheshwari A, Lip GYH et al. Relation of the CHA(2)DS(2)-VASc score to risk of thrombotic and embolic stroke in community-dwelling individuals without atrial fibrillation (from the Atherosclerosis Risk in Communities [ARIC] study). Am J Cardiol 2019;123:402–408. 30527796 10.1016/j.amjcard.2018.10.037 PMC6424356

[104] Mitchell LB, Southern DA, Galbraith D, Ghali WA, Knudtson M, Wilton SB. Prediction of stroke or TIA in patients without atrial fibrillation using CHADS_2_ and CHA_2_DS_2_-VASc scores. Heart 2014;100:1524–1530. 24860007 10.1136/heartjnl-2013-305303

[105] Freeman JV, Shrader P, Pieper KS, Allen LA, Chan PS, Fonarow GC et al. Outcomes and anticoagulation use after catheter ablation for atrial fibrillation. Circ Arrhythm Electrophysiol 2019;12:e007612. 31830822 10.1161/CIRCEP.119.007612

[106] Granger CB, Alexander JH, Mcmurray JJV, Lopes RD, Hylek EM, Hanna M et al. Apixaban versus warfarin in patients with atrial fibrillation. N Engl J Med 2011;365:981–992. 21870978 10.1056/NEJMoa1107039

[107] Patel MR, Mahaffey KW, Garg J, Pan G, Singer DE, Hacke W et al. Rivaroxaban versus warfarin in nonvalvular atrial fibrillation. N Engl J Med 2011;365:883–891. 21830957 10.1056/NEJMoa1009638

[108] Connolly SJ, Ezekowitz MD, Yusuf S, Eikelboom J, Oldgren J, Parekh A et al. Dabigatran versus warfarin in patients with atrial fibrillation. N Engl J Med 2009;361:1139–1151. 19717844 10.1056/NEJMoa0905561

[109] Kirchhof P, Camm AJ, Goette A, Brandes A, Eckardt L, Elvan A et al. Early rhythm-control therapy in patients with atrial fibrillation. N Engl J Med 2020;383:1305–1316. 32865375 10.1056/NEJMoa2019422

[110] Mathew JS, Marzec LN, Kennedy KF, Jones PG, Varosy PD, Masoudi FA et al. Atrial fibrillation in heart failure US ambulatory cardiology practices and the potential for uptake of catheter ablation: an National Cardiovascular Data Registry (NCDR^®^) Research to Practice (R_2_P) project. J Am Heart Assoc 2017;6:e005273. 28862932 10.1161/JAHA.116.005273 PMC5586408

[111] Quiroz JC, Brieger D, Jorm LR, Sy RW, Falster MO, Gallego B. An observational study of clinical and health system factors associated with catheter ablation and early ablation treatment for atrial fibrillation in Australia. Heart Lung Circ 2022;31:1269–1276. 35623999 10.1016/j.hlc.2022.04.049

[112] Russo AM, Zeitler EP, Giczewska A, Silverstein AP, Al-Khalidi HR, Cha Y-M et al. Association between sex and treatment outcomes of atrial fibrillation ablation versus drug therapy: results from the CABANA trial. Circulation 2021;143:661–672. 33499668 10.1161/CIRCULATIONAHA.120.051558 PMC8032462

[113] Ngo L, Ali A, Ganesan A, Woodman RJ, Adams R, Ranasinghe I. Utilisation and safety of catheter ablation of atrial fibrillation in public and private sector hospitals. BMC Health Serv Res 2021;21:883. 34454482 10.1186/s12913-021-06874-7 PMC8400841

