# Supplementary material for: Freedom from atrial arrhythmia and other clinical outcomes at 5 years and beyond after catheter ablation of atrial fibrillation: a systematic review and meta-analysis
Source: Eur Heart J Qual Care Clin Outcomes. 2023 Jun 19;9(5):447–58. doi: 10.1093/ehjqcco/qcad037 (PMC10658515; doi:10.1093/ehjqcco/qcad037)
Supplement: qcad037_Supplemental_File [file qcad037_supplemental_file.docx]

**Very long-term outcomes after catheter ablation of atrial fibrillation: A systematic review and meta-analysis**

**Authors:**

**Supplemental Tables**

Table S1: Literature search keywords

Table S2: Summary of included studies

Table S3: Quality assessment of individual studies

Table S4: Comparison of results with arcsine and logit transformation

**Supplemental Figures**

Figure S1: Funnel plots of the meta-analysis of freedom from atrial arrhythmia recurrence after single procedure

Figure S2: Funnel plots of the meta-analysis of freedom from atrial arrhythmia recurrence after multiple procedures

Figure S3: Funnel plots of the meta-analysis of all-cause mortality

Figure S4: Funnel plots of the meta-analysis of stroke or transient ischaemic attack

Figure S5: Funnel plots of the meta-analysis of major bleeding

Figure S6: Leave-one-out analysis for the meta-analysis of freedom from atrial arrhythmia recurrence at 5-years after a single procedure

Figure S7: Bias-corrected trim-and-fill funnel plot for meta-analysis of freedom from atrial arrhythmia recurrence at 5-years after a single procedure

**Table S1: Literature search keywords**

| **Database** | **Keywords** | | |
| --- | --- | --- | --- |
|  | **Patient/population** | **Intervention** | **Outcomes** |
| Pubmed | Atrial fibrillation[mh] OR atrial fibril*[tiab] OR auricular fibril*[tiab] OR atrium fibril*[tiab] | Catheter ablation[mh] OR catheter ablati*[tiab] OR pulmonary vein isolation*[tiab] | (long term[tiab] OR long-term[tiab]) AND (Treatment outcome[mh] OR treatment outcome*[tiab] OR recrudescen*[tiab] OR reoccurren*[tiab] OR success*[tiab] OR treatment failure*[tiab] OR disease free[tiab] OR stroke[mh] OR stroke*[tiab] OR heart failure[tiab] OR heart failure[mh] OR haemorrhage[tiab] OR hemorrhage[tiab] OR hemorrhage[mh] OR cerebrovascular accident[tiab]) |
| Embase | ‘heart atrium fibrillation’:de OR ‘atrium fibrillation’:ti,ab OR ‘atrial fibrillation’:ti,ab OR ‘auricular fibrillation’:ti,ab | ‘catheter ablation’:de OR ablati*:ti,ab OR ‘ablation therapy’:de OR ‘pulmonary vein isolation’:ti,ab | (‘long term’:ti,ab OR ‘long-term’:ti,ab) AND ('recurrent disease':de,ab,ti OR recurren*:ti,ab OR relaps*:ab,ti OR recrudescen*:ab,ti OR reoccurren*:ab,ti OR 'treatment outcome':de OR outcome*:ab,ti OR 'treatment failure':de,ab,ti OR 'treatment failures':ab,ti OR success*:ab,ti OR 'disease free':ab,ti OR ‘cerebrovascular accident’/exp OR ‘cerebrovascular accident’:ab,ti OR ‘stroke’:ab,ti OR ‘heart failure’/exp OR ‘heart failure’:ab,ti OR ‘bleeding’/exp OR ‘bleeding’:ab,ti OR ‘haemorrhage’:ab,ti OR ‘hemorrhage’:ab,ti) |

**Table S2: Summary of included studies**

| First author (year) | Design | Retrospective | Location | Ablation energy | Population | Ablation strategy | Enrolment period | Mean follow-up time (SD, in years) | Definition of atrial arrhythmia recurrence and blanking period | Follow-up strategy |
| --- | --- | --- | --- | --- | --- | --- | --- | --- | --- | --- |
| Nademanee (2008)^1^ | Obs | N | US | RFA | ≥65y patients with ≥1 risk factors for stroke | PVI + CFAE | 2000-2006 | 2.3 ± 1.7 | - Patient symptoms in conjunction with follow-up ECG or event monitor recording and annual Holter monitoring.  - 3 months of blanking period. | Follow-up at arrhythmia clinic every 3 months. |
| Bhargava (2009)^2^ | Obs | N | US, Canada, and Italy | RFA | Patients with symptomatic, drug-resistant AF who aged 18-85y | PVI + SVC isolation + LA posterior wall isolation + non-PV triggers ablation | 2000-2006 | 4.7 ± 1.3 | - Arrhythmia recurrence was not clearly defined.  - 8 weeks of blanking period. | 48h Holter monitoring after discharge at 3, 6, 9, and 12 months after the procedure. Longer term follow-up was performed by the referring doctor and confirmed only with phone calls. |
| Sawhney (2009)^3^ | Obs | Y | US | RFA | Patients with PAF undergoing PVI at a single centre with at least five years of follow-up data | PVI + CTI ablation | 2002-2003 | 5.3 ± 0.4 | - Arrhythmia recurrence was not clearly defined.  - 3 months of blanking period. | ECG and symptoms at outpatient clinic at 1, 3, 6 and 12 months after the procedure and every 6 months afterwards.  Mobile outpatient telemetry monitoring for any reported symptoms of arrhythmia recurrence.  Telephone visit at the end of five-year follow-up period. |
| Hunter (2010)^4^ | Obs | N | UK | RFA | All consecutive patients undergoing CA of AF. | PVI + linear ablation for PeAF + CTI ablation in case with Aflutter + CFEA | 2002-2007 | 4.3±3.8 | - Symptomatic and/or documented AF/AT lasting >30s after 3-month blanking period. | Patients were followed-up at 3 months and again at 6 months if symptomatic initially, with a period of ambulatory monitoring of 2-7 days. Further monitoring prompted by symptoms with ECG or through telephone. |
| Tzou (2010)^5^ | Obs | N | USA | RFA | Consecutive patients undergoing PVI at University of Pennsylvania. | PVI + non-PV triggers ablation | 2001-2003 | 5.9±1.5 | Atrial arrhythmia recurrence and blanking period not clearly defined. | Outpatient visits at 6 weeks, 6 months, 1 year, and at least annually thereafter.  4-weeks of trans-telephonic monitoring at 3 to 9 months post-ablation for consideration of drug cessation. |
| Daly (2011)^6^ | Obs | Y | New Zealand | RFA | All patients who underwent percutaneous radiofrequency ablation at Christchurch Hospital identified from the catheter laboratory log. | PVI +/- CTI ablation +/- left atrial roof line/mitral isthmus line ablation +/- substrate ablation | 2001-2009 | 3.0±0.9 | Both arrhythmia recurrence and blanking period were not clearly defined. | Rhythm outcome was derived from a combination of patient ECGS, Holter monitor results, and clinical notes. |
| Fiala (2012)^7^ | Obs | Y | Czech Republic | RFA | 866 consecutive patients who underwent catheter ablation for symptomatic AF. | PVI +/- LA roof mitral isthmus linear ablation | 2003-2011 | 4.1±2.2 | - ECG documented AF/AT, and any history of palpitations suggestive of AF/AT lasting > 30 seconds.  - Blanking period was not clearly defined. | Patients were seen at the outpatient department after 6 weeks, 3, 6, 9, and 12 months, and then every 6 months in the first post-ablation years. |
| Hunter (2012)^8^ | Obs | N | UK & Australia | RFA | A multicentre registry including centres in the UK and Australia. | PVI + CEFA | NR | 4.6±6.4 | - Any documented AF/AT lasting ≥30s after a blanking period of 3 months. | Follow-up after 6 months included clinic visits, telephone appointments, follow-up with local cardiologists and open access to arrhythmia nurse specialists. |
| Sorgente (2012)^9^ | Obs | N | USA | RFA | 103 of 249 consecutive patients who underwent PVI at Beth Israel Deaconess Medical Center prospectively followed for >=4 years after the index procedure. | PVI + LA ablation lines (roof line, mitral isthmus line, posterior LA line) | 2002-2006 | 6.0±1.8 | - An asymptomatic or symptomatic atrial tachyarrhythmia consistent with AF or AT was documented to last >10 seconds.  - A blanking period of 30 days. | Evaluation of symptomatic or asymptomatic AF or AT was performed using a 1- to 3-week continuous loop event recorder at 1, 3, 6, and 12 months, and then every 6 or 12 months. |
| Neumann (2013)^10^ | Obs | N | Germany | CRYO | Patients with symptomatic, drug refractory PAF | PVI | 2005-2007 | NR | Both arrhythmia recurrence and blanking period were not clearly defined. | Quarterly in the first year and annually afterwards, during which 7-day Holter ECG was performed.  ECG performed in case of any palpitations. |
| Uchiyama (2013)^11^ | Obs | Y | Japan | RFA | Patients with symptomatic, drug refractory PAF and 5-year follow-up data | PVI + LA posterior wall ablation ± CTI ablation | 2003-2006 | 6.4 ± 1 | - Arrhythmia lasting >30s (with or without AAD) or symptoms suggestive of arrhythmia recurrence.  - No pre-defined blanking period. | Follow-up visits at 3, 6, 9, and 12 months and later, by referral.  Clinical interview, ECG and 24h Holter ECG. |
| Wang (2013)^12^ | Obs | N |  | RFA | Patients with symptomatic, drug refractory PAF | PVI + SVC isolation ± CTI ablation ± mitral isthmus ablation | 2005-2007 | 5.2 ± 1.1 | - Atrial arrhythmia lasting >30s without AAD.  - 3 months of blanking period. | Follow-up at least five years through telephone interview.  12-lead ECG or 24h Holter ECG every 3-6 months after ablation was encouraged. |
| Wojcik (2013)^13^ | Obs | N | Germany | CRYO | Patients aged 18-80y with symptomatic, drug refractory AF | PVI ± mitral isthmus ablation ± roof line ablation ± CFAE ablation | NR | 5.1 ± 2.6 | - First documented AF/flutter, atrial tachycardia episode lasting >30s.  - 3 months of blanking period. | Quarterly in the first year and annually afterwards, during which 7-day Holter ECG and 12-lead ECG were obtained. |
| Zhou (2013)^14^ | Obs | Y | China | RFA | Consecutive patients with nonparoxysmal AF who underwent radiofrequency catheter ablation for the first time. | PVI + Roof line ablation + CFAE ablation | 2005-2009 | 4.2±0.8 | - Any episodes of AF/AT >30s during follow-up after 3-months of blanking period. | Through outpatient visit once a month for 1 year and then every 6 months or at any time the patient had tachycardia-related symptoms. |
| Gaita (2014)^15^ | Obs | N | Italy | RFA | Patients undergoing AF ablation in a single centre | PAF: PVI  Persistent AF: PVI + linear ablation + CFAE ablation from 2004 | 2001-2009 | 5.1 ± 2.6 | - A sustained AF/flutter lasting >30s either symptomatically or recorded by ECG or 24h Holter.  - 3 months of blanking period. | Follow-up during ambulatory visits (performed at 1, 3, 6 months, and then yearly) or by telephone |
| Gal (2014)^16^ | RCT | N | Netherlands | RFA | Consecutive patients with symptomatic AF | Conventional PVI or Multi-electrode, duty-cycled, phased RF ablation | NR | 3.7 ± 1.8 | - An ECG showing the characteristics of AF, or on a 30s telemetry strip.  - 3 months of blanking period. | Follow-up visits scheduled at 3, 6, and 12 months after procedure (including 24h Holter ECG), and by referral afterwards. |
| Hayashi (2014)^17^ | Obs | N | Japan | RFA | Patients with paroxysmal or persistent AF referred for catheter ablation at a single centre | PVI + CTI ablation | 2002-2008 | 5.8 ± 1.7 | - A symptomatic episode lasting >30s confirmed by ECG.  - 1 month of blanking period. | Patients were routinely monitored by clinical examination, 12-lead ECG for 3 min and 24h Holter ECG at 1, 3, 6 months and every 3 months thereafter. |
| Takigawa (2014)^18^ | Obs | N | Japan | RFA | Patients referred for catheter ablation due to symptomatic, drug refractory PAF | PVI ± CTI ablation ± non-PV foci ablation | 2003-2009 | 2.5 ± 2.8 | - AT lasting >30s, off AADs.  - 1 month of blanking period. | Patients were prospectively followed-up at 2, 6, 10, 14, 24, 36, and 48 weeks after the procedure, with 12-lead ECG at each visit and Holter monitoring every 3 months. |
| Costa (2015)^19^ | Obs | N | Portugal | RFA | All consecutive patients with symptomatic drug-refractory AF undergoing percutaneous PVI in two Portuguese centres. | PVI + CTI ablation | 2005-2011 | 2.4 ±1.7 | - Symptomatic or documented AF lasting >30s after a blanking period of 3 months. | Follow-up was done by outpatient visits with 12-lead ECG and 24h Holter at 1, 3, 6 and 12 months in the first year, and yearly thereafter. |
| Karasoy (2015)^20^ | Obs | Y | Denmark | RFA | Using Danish administrative registries |  | NR | 3.7 ±2.7 | - Any hospitalisation for AF with or without direct cardioversion, or re-ablation procedure after a blanking period of 3 months. | Follow-up was done through data linkage within the registry. |
| Noseworthy (2015)^21^ | Obs | Y | USA | NR | Patients from the Optum Labs Data Warehouse, which includes privately insured and several Medicare Advantage enrolless in USA. |  | 2005-2012 | NR | Not applicable (primary outcome was stroke or TIA) | Follow-up was done through data linkage. |
| Schreiber (2015)^22^ | Obs | Y | Germany | RFA | A total of 549 patients with persAF underwent CA using the stepwise approach at our institution | PVI + Linear ablation | 2007-2009 | 4.9 ± 1.3 | - Any AT/AF episode lasting >30 s in sequential Holter ECG, device interrogation, or tele-ECGs  - 3 months of blanking period. | Patients were followed clinically every 3 to 6 months with detailed symptom evaluation and a 12-lead ECG. Holter ECGs were performed at least every 3 months for the first year after CA and afterward every 6 months. |
| Tran (2015)^23^ | Obs | N | Switzerland | RFA | Every patient who underwent first AF ablation from January at a single centre. | PVI + Linear LA ablation for non-paroxysmal AF ± CTI ablation | 2002-2005 | 9.1 ± 0.5 | - A run of AF, atrial flutter, or AT > 30s.  - 3 months of blanking period. | Rhythm monitoring was advised at 1 month, 3 months, 6 months, 1 year, and 2 years. |
| Yamaguchi (2015)^24^ | Obs | Y | Japan | RFA (radiofrequency hot balloon) | All patients who had AF ablation from 2002-2005 in a single centre | PVI + LA posterior wall ablation | 2006-2009 | 6.2 ± 1.0 | - Symptomatic and/or asymptomatic episodes of ATAs lasting >30s and identified on 12-lead surface ECG, mobile and/or 24h Holter ECG.  - This study did not adhere to a predefined blanking period. | One day after the procedure, a 12-lead surface ECG and a 24h Holter ECG were performed and repeated after 3, 6, and 12 months.  Clinical follow-up was regularly carried out every 6 months at the outpatient clinic, performing a 12lead surface, mobile ECG and 24-hour Holter ECG during at least 5 years. |
| Bunch (2016)^25^ | Obs | Y | USA | NR | Patients ≥18y underwent their index AF ablation at LDS Hospital (Salt Lake City, UT) or Intermountain Medical Center (Murray, UT) and had at least 5 years of follow-up. | Not clearly reported. | NR | 5.9 ± 1.4 | - Arrhythmia recurrence was not clearly defined.  - 3 months of blanking period. | Atrial flutter or AF recurrences were documented through review of the AF ablation registry with ambulatory heart monitors performed at 3, 6, 9, and 12 months post-ablation and then thereafter based upon recurrence of clinical symptoms |
| Teunissen (2016)^26^ | Obs | N | Netherlands | RFA | All consecutive patients suffering from symptomatic, drug-refractory, or drug-intolerant AF who underwent primary PVAI in the University Medical Center Utrecht. | PVI ± CTI ablation | 2005-2011 | 5.5 ± 1.9 | - The occurrence of AF, atrial flutter, or AT of > 30s duration, documented by an ECG or device recording system.  - 3 months of blanking period. | Patients were seen at the outpatient clinic at 3, 6, 12, and 24 months after the procedure. Rhythm status was evaluated using patient’s history and a 12-lead ECG in every visit and with additional 48-h Holter recordings at 3 and 6 months. |
| Hung (2017)^27^ | Obs | NR | Taiwan | RFA | Consecutive patients who underwent catheter ablation of drug-refractory, symptomatic AF in Taipei Veteran General Hospital (VGH). | PVI + CTI ablation + Linear ablation + CFAE ablation + non-PV triggers ablation | 2003-2011 | 2.4±1.9 | - An episode of atrial arrhythmia lasting >30s.  - Blanking period was not clearly defined. | Patients were followed-up every 1-3 months at outpatient clinic for a year and every 6 months thereafter. |
| Kawaji (2017)^28^ | Obs | Y | Japan | RFA | Patients undergoing AF ablation in a single centre | PVI ± linear ablation ± CTI ablation ± SVC isolation | 2004-2015 | 5.0 ± 2.5 | Documented AF and/or AT lasting for >30s or those requiring repeat ablation procedures.  3 months of blanking period. | A 12-lead electrocardiogram was routinely measured at each clinical visit and 24-hour Holter monitoring was recommended at 3-, 6-, 12-month and yearly thereafter. |
| Miyazaki (2016)^29^ | Obs | Y | Japan | RFA | The study consisted of 135 consecutive patients who underwent catheter ablation of persistent AF using a biatrial linear defragmentation approach | PVI ± Linear ablation ± CFAE | 2010-2011 | 4.2 ± 2.3 | Documented episodes of AF lasting >30s.  3 months of blanking period. | Patients underwent continuous ECG monitoring as inpatients for 3 days after the procedure. A 24h Holter recording was obtained at 1, 3, and 6 months after ablation and thereafter at 6-month intervals. |
| Nielsen (2017)^30^ | RCT | N | Multicentre RCT | RFA | Patients with symptomatic PAF | PVI ± Linear ablation ± CTI ablation |  | NR | The primary study end points were the burden of AF (defined as the percentage of time in AF on each Holter recording) and the cumulative burden of AF (defined as the percentage of time in AF on all the Holter recordings obtained during follow-up). Only episodes of AF longer than 1 minute were included in the analysis. | Clinical follow-up and a 7-day Holter-monitor recording were scheduled at 3, 6, 12, 18, and 24 months.  The 5-year follow-up included clinical follow-up with recording of the ECG, current medication, and ablations performed after the 2-year follow-up visit, and one 7-day Holter recording. |
| Saliba (2017)^31^ | Obs | Y | Israel | NR | Patients identified in the Clalit Health Services database (a health care provider). | Not described | 2005-2015 | NR | Not applicable as the primary outcome was stroke/TIA. | Follow-up was done by data linkage. |
| Winkle (2017)^32^ | Obs | Y | USA | RFA | The subjects were consecutive symptomatic patients undergoing AF ablation at Sequoia Hospital, Redwood City, CA. | PVI + CTI ablation + mitral isthmus ablation + Low posterior LA lines ablation + LA CFAE ablation + Ablation at the CS region | 2003-2015 | NR | AF, atrial flutter, or AT lasting >30s after a 3-month blanking period. | Patients transmitted daily electrocardiogram (ECG) strips for 1–3 months post-ablation and were seen at 3 months when echocardiogram and ambulatory ECG monitoring for !24 hours to 14 days were performed. |
| Yagishita (2017)^33^ | Obs | N | Japan | RFA | Consecutive patients undergoing catheter ablation of persistent AF at Musashino Red Cross Hospital, Tokyo. | PVI ± Linear ablation ± CTI ablation |  | 3.5 ± 2.5 | Any episode of AF or AT lasting ≥30s.  3 months of blanking period. | The follow-up visits consisted of a clinical interview, ECG, and 24-hour Holter monitoring every 3, 6, and 12 months. After 12 months, the patients were seen thereafter every 6-12 months at our centre. |
| Yin (2017)^34^ | Obs | Y | China | RFA | Patients with symptomatic refractory PeAF undergoing their first catheter ablation at the First Affiliated Hospital of Dalian Medical University. | PVI + LA roof line ablation + complex rapid and fractionated electrograms ablation + ablation from the endocardium of the CS + additional linear LA ablation | 2009-2012 | 3.8±1.5 | AF or any sustained atrial arrhythmia after the last ablation procedure, after 3 months of blanking period. | After a 3-month blanking period of ablation, patients were reappraised at 3, 6, 9, and 12 months and every 6 months thereafter, up to 3 years. |
| Akkaya (2018)^35^ | Obs | N | Germany | CRYO | Patients undergoing catheter ablation for symptomatic AF | PVI | 2012 | NR | AF or AT episode lasting >30s without AAD.  3 months of blanking period. | Patients were monitored via resting ECG, 7-day Holter-ECG, and echocardiography during follow-up visits at 3- or 6-month intervals in the first year, and then once per year. |
| Ang (2018)^36^ | Obs | N | UK | COMBINED | Patients undergoing catheter ablation of PAF at a single institution as part of an RCT comparing outcomes after different types of ablation energy (RF, CRYO, and RF+CRYO) | PVI | 2008-2014 | 5.0 ± 2.1 | Documented AF/AT  lasting > 30s.  3 months of blanking period. | Patients were followed up at 3, 6 and 12 months with a 7-day Holter recording. There was open access to arrhythmia nurse specialists subsequently and further ambulatory monitoring prompted by symptoms. |
| Chelu (2018)^37^ | Obs | Y | Netherlands | RFA | Patients who underwent LGE-MRI of sufficient quality for LA fibrosis assessment, and subsequently underwent AF ablation in a registry. | PVI ± LA posterior wall ablation ± CTI ablation | 2006-2010 | 1.5 ± 1.9 | Documented rhythm of AT/AF/atrial flutter or a repeated ablation after the blanking period.  3 months of blanking period. | Documentation of recurrence and repeated ablation was obtained through chart review. |
| De Greef (2018)^38^ | Obs | N | Belgium | COMBINED | Patients with symptomatic, drug resistant recurrent AF with no or limited structural heart disease | PVI | 2006-2014 | 3.7 ± 1.8 | Documented AF>30 s after a single procedure, without AADs.  1 month of blanking period. | Follow-up included schedule visits every 7 months and unscheduled visits if symptomatic. In case of symptoms, the related arrhythmia was documented either by ECG, Holter-monitoring (1 to 7-day) or event-recording. |
| De Maat (2018)^39^ | Obs | Y | Netherlands | RFA | All patients scheduled for a first PVI between 2003 and 2013 at the University Medical Center Groningen, the Netherlands. | PVI ± linear ablation | 2003-2013 | 3.8 ± 2.7 | AF, atrial flutter or other atrial arrhythmias lasting >30 s, without use of AADs.  3 months of blanking period. | Patients visited our clinic at 3, 6 and 12 months, and annually thereafter. At each visit a routine 12-lead ECG was performed, and when atrial arrhythmia was detected, a 12-lead rhythm strip (>30 s) was recorded. |
| Fredersdorf (2018)^40^ | Obs | Y | Germany | RFA | Consecutive patients with symptomatic AF treated with pulmonary vein isolation (PVI) using a PVAC in a low-volume centre. | PVI | 2009-2012 | 4.3±1.2 | Either symptoms consistent with AF or any documented episode of AF or AT/ atrial flutter lasting >30s after 3 months of blanking period. | All patients were evaluated AT 3, 6 and12months following the ablation procedure in our clinic. Long-term follow-up was performed at the time point of the study between 23 and 77 months. |
| Gaita (2018)^41^ | Obs | N | Italy | RFA | Consecutive patients undergoing AF ablation | PVI ± linear ablations ± CFAE ablation | 2004-2006 | 10.4 ± 0.6 | ECG-documented sustained AF, atypical flutter or AT lasting >30s.  Blanking period not defined. | Recurrences were detected by routine ambulatory visits (performed at 1, 3, 6 months and then twice a year), with collection of patients’ characteristics, blood samples, symptoms and 24h Holter ECG recordings. |
| Kim (2018)^42^ | Obs | Y | Korea | NR | Consecutive patients with AF and more than 2 years of follow-up. | PVI + linear ablation + CFAE ablation | 2000-2013 | NR | Any atrial arrhythmias lasting >30s after a 3-month blanking period. | After ablation, patients were asked to visit the outpatient clinic at 1, 3, 6, and 12 months and then every 6 months thereafter or whenever they experienced tachycardia-related symptoms. |
| Lee (2018)^43^ | RCT | N | Korea | RFA | This study was a follow-up of a randomized single-center trial that evaluated for 1-year clinical outcome. | PVI + non-PV triggers ablation | 2008-2013 | NR | Any atrial tachy-arrhythmia lasting >30s.  Blanking period of 3 months. | Ambulatory monitoring and daily 12-lead ECGs were applied to patients for 1 to 2 days in the hospital. Patients were asked to visit the outpatient clinic at 1, 3, 6, 9, and 12 months after the procedure and then every 6 months thereafter. |
| Reissmann (2018)^44^ | Obs | Y | Germany | LASER | Patients with drug-refractory PAF who underwent VGLB-based PVI | PVI | 2009-2013 | 4.8 ± 1.7 | Any AT (> 30 s. duration), either anamnestic (in case of symptoms suggestive of arrhythmia recurrence) or documented on Holter ECG and/or 12-lead ECG.  3 months of blanking period. | Patients completed outpatient clinic visits at 3, 6, and 12 months including Holter ECGs. Thereafter, clinical follow-up visits at our outpatient clinic or at the referring physician were recommended in 6-month intervals. |
| Tilz (2018)^45^ | Obs | N | Germany | RFA | The study prospectively enrolled consecutive patients with symptomatic, drug refractory PAF undergoing CPVI at St. Georg’s hospital in Hamburg, Germany. | PVI | 2003-2004 | 10.7 ± 0.5 | Symptomatic or asymptomatic episodes of AF/ AT lasting >30s.  This study did not adhere to a predefined blanking period. | One day after the procedure, a 12-lead ECG, a transthoracic echocardiogram, and a 24h Holter ECG were performed. Clinical follow-up was regularly performed after 1, 3, 6, and 12 months, as well as every 6 months (≤5 years) and every year (>5 years) at the outpatient clinic or at the referring physician performing 12-lead ECG and 24h Holter ECG. At the 10-year follow-up time point, a 72-hour  Holter-ECG was performed. |
| Canpolat (2019)^46^ | Obs | N | Turkey | CRO | Patients who underwent index PVI alone using CB2 for symptomatic paroxysmal or persistent AF at a single centre. | PVI | 2013-2017 | 3.4±1.9 | Any episodes of atrial tachyarrhythmia lasting >30s after the 3-month blanking period. | Patients were scheduled for outpatient clinic visits at 1st, 3rd, 6th, and 12th months and every 1 year thereafter. |
| Efremidis (2019)^47^ | Obs | Y | Greece | RFA | Consecutive patients with drug refractory AF who underwent PVI | PVI ± CFAE ablation | 2009-2015 | 3.3 ± 1.7 | Documented symptomatic or asymptomatic AF or AT episodes lasting >30s.  3 months of blanking period. | 24 or 48 h Holter recordings and 12-lead ECG were performed in all patients at 1, 3, 6, 9 and 12 months post-ablation and every 6 months thereafter. |
| Gedikli (2019)^48^ | Obs | N | US | RFA | Consecutive AF patients undergoing RF catheter ablation at a single centre who had undergone preprocedural TEE. | PVI + LA posterior wall isolation ± non-PV triggers ablation or isolation | 2009-2010 | 6.6 ± 2 | NA | They were monitored for TE complications by follow-up TEE, clinic visits, and phone calls by our research staff at 6-month intervals for 24 months and annually thereafter. |
| Kim (2019)^49^ | Obs | Y | Korea | RFA | Patients included in the Yonsei AF Ablation Cohort Database | PVI ± Linear ablation ± SVC isolation ± CFAE ablation | NR | 5.9 ± 0.2 | Any episode of AF or AT lasting for at least 30 s.  3 months of blanking period. | All patients underwent ECG during every outpatient clinic visit and 24-hour Holter recording at 3 and 6 months and then every 6 months thereafter. |
| Kornej (2019)^50^ | Obs | Y | Germany | RFA | Consecutive high-symptomatic patients presenting for the catheter ablation from The Leipzig Heart Center AF Ablation Registry. | PVI + linear ablations (LA roof, basal posterior wall, mitral isthmus) | 2007-2011 | NR | Atrial arrhythmia recurrence and blanking period were not clearly defined. | All patients were followed in the outpatient clinic after catheter ablation. During the follow-up period, 7-days Holter ECG recordings were performed (immediately, 3, 6 and 12 months after the ablation, then every 6 months). |
| Lin (2019)^51^ | Obs | Y | Taiwan | RFA | A nation-wide population-based cohort study using data from the Taiwan National Health Insurance Research Database (NHIRD). | Not described. | 2001-2010 | 4.3±2.8 | Recurrence was defined as either (1) recurrence of original arrhythmia or (2) receipt of a second RFCA during the follow-up period.  Blanking period not defined. | Follow-up was done through data linkage. |
| Packer (2019)^52^ | RCT | N | Multicentre RCT | RFA+CRYO | Eligible patients were aged 65 years and older or younger than 65 years with 1 or more risk factors for stroke (hypertension, heart failure, history of stroke, diabetes, or other heart roblems), had 2 or more episodes of paroxysmal AF or 1 episode of persistent AF in the prior 6 months, and were suitable for catheter-based treatment or rhythm and/or rate control drug therapy. Patients were excluded if they had a prior left atrial catheter ablation for AF or had failed 2 or more antiarrhythmic drugs. | PVI + CFAE ablation + ganglia ablation + linear ablation | 2009-2016 | 3.9±2.0 | A 30-s episode of AF confirmed through blinded review by an ECG Core Laboratory Committee.  Blanking period of 3 months. | Scheduled patient follow-up occurred at 3, 6, and 12 months and then every 6 months thereafter. |
| Baba (2020)^53^ | Obs | Y | Japan | RFA | Consecutive patients referred to Ibaraki Prefectural Central Hospital for initial catheter ablation of paroxysmal AF. | PVI + CTI ablation + non-PV triggers ablation | 2012-2017 | NR | Atrial arrhythmias lasting >30s after the 3-month blanking period. | The first outpatient clinic visit with 12-lead ECG recording was scheduled around 2 weeks after the procedure and then every 2 months thereafter. |
| Baek (2020)^54^ | Obs | Y | Korea | RFA | Patients who underwent radiofrequency catheter ablation for symptomatic drug-refractory AF at a single tertiary hospital. | PVI ± non-PV triggers ablation ± CFAE | 2004-2016 | 4.8 ± 3.2 | Any episode of AF plus AT lasting for ≥30 s.  3 months of blanking period. | Ambulatory monitoring and daily 12-lead electrocardiogram (ECG) were applied to patients at 1 to 2 days after AF ablation. Patients were asked to visit our outpatient clinic 1, 3, 6, and 12 months after catheter ablation and every 6 months thereafter, or whenever symptoms occurred. |
| Heeger (2020)^55^ | Obs | Y | Germany | CRYO | All patients referred to two electrophysiology centers in Hamburg, Germany (Asklepios Klinik St. Georg and Asklepios Klinik Harburg). | PVI | 2012-2014 | 4.9 ± 1.6 | Symptomatic and/or documented episodes of AF/AT lasting > 30s.  3 months of blanking period. | Patients completed outpatient clinic visits at 3, 6, 12 and in 6months intervals thereafter. Telephone interviews were conducted in all patients, and 12‑lead ECGs and/or 24 h-Holter-ECGs completed in case of symptoms suggestive of recurrence of AF/AT. |
| Kis (2020)^56^ | Obs | Y | Netherlands | RFA+CRYO | All consecutive individuals who had undergone a CA of AF and completed 4-years of follow-up | PVI + CFAE ablation + linear lines ablation | 2001-2016 | 6.1 ± 1.6 | Sinus rhythm could not be maintained despite repeated ablation or AAD. Outcomes were tracked from medical records. | Retrospective study using medical records. |
| Kriatselis (2020)^57^ | Obs | N | Germany | RFA | Patients with symptomatic persistent AF scheduled for PVI. | PVI | 2007-2010 | 7.2 ± 1.1 | Any atrial arrhythmia (with the exception of typical flutter) with a duration of more than 30 s by ECG and/or ambulatory cardiac rhythm monitoring.  3 months of blanking period. | The patients were seen at our outpatient department at 3 months, 6 months, and then every 6 months after the last ablation procedure. During each visit, an ECG and 24-h Holter monitoring were obtained. |
| Romero (2020)^58^ | Obs | N | USA | RFA | Consecutive patients undergoing their first RFA procedure for non-paroxysmal AF with completed 5-year follow-up. Patients who underwent ablation of complex fractionated atrial electrograms and empirical lines were excluded. | PVI ± LA posterior wall isolation ± CS and SVC isolation | 2010-2014 | NR | >30s of any atrial arrhythmia (ie, AF, atrial flutter, and AT).  3 months of blanking period. | Patients were routinely evaluated at the arrhythmia centre 6-week post-ablation, followed by 3, 6, and 12-month intervals, and then annually for the remaining follow-up period. |
| Sawhney (2020)^59^ | Obs | N | USA | CRYO | Consecutive patients undergoing cryoablation for persistent AF across three UK and eight European centres. | PVI | 2011-2017 | 2.5±2.2 | Recurrence of AF or AT lasting ≥30s on ECG monitoring after the 3-month blanking period. | Routine follow-up was organized as per the standard clinical practice at each centre. Typically, patients were reviewed in outpatient clinics at 3, 6, and 12 months post-index cryoablation procedure. Further periods of monitoring were dictated by symptoms or suspicion of arrhythmia recurrence. |
| Sugumar (2020)^60^ | Obs | N | Australia | RFA | Consecutive patients who underwent >1 CA for persistent AF at 4 high-volume tertiary centres (Alfred Health; Cabrini Hospital; Melbourne Private Hospital and Royal Melbourne Hospital; Melbourne Australia). | PVI ± LA posterior wall isolation ± Linear ablation ± CFAE ablation ± non-PV triggers ablation | 2004-2018 | 3.8 ± 2.7 | Any documented  atrial arrhythmia lasting >30s after the last ablation procedure.  3 months of blanking period. | Follow-up was performed through clinic visits and/or telehealth consult. Patients were reviewed in clinic at 6 weeks and then at 6-month reviews and were asked to contact the dedicated arrhythmia nurse or treating physician if recurrent symptoms occurred in the interim. Follow-up rhythm was confirmed via pre-existing cardiovascular implantable electronic devices where available, electrocardiograms, and 24h Holter monitors. |
| Ding (2021)^61^ | Obs | Y | Korea | NR | This study used the national health claims established by the National Health Insurance Service (NHIS) of Korea. | NA | 2006-2015 | NR | The primary endpoint was AF recurrence after index ablation which was determined using surrogate markers of cardioversion or repeat AF ablation. | Follow-up was done through data linkage |
| Esato (2021)^62^ | Obs | N | Japan | RFA | This study used data from the Fushimi AF registry, a community-based prospective survey of patients with AF who visited the participating medical institutions in Fushimiku, Kyoto, Japan. | PVI | 2011-2019 | 4.7 ± 3.6 | NA.  The clinical endpoints in this analysis were the incidences of major adverse cardiovascular events and all-cause death during the follow-up period. | NA |
| Gallagher (2021)^63^ | RCT | N | United Kingdom | COMBINED | All patients with persistent AF scheduled for a first left atrial ablation were randomly assigned 1:1 to either a standard RF method or to dual catheter cryotherapy; randomization occurred on the day of the procedure. | PVI ± CTI ablation |  | 3.5 ± 1.5 | Any episode of AF/AT >30 s, either  symptomatic or asymptomatic, documented on a Holter ECG and/or 12-lead resting ECG or other ECG recording devices.  6 months of blanking period. | Follow-ups were scheduled at 3-, 6-, and 12-month post-ablation, then at 3 and 5 years, with attendance at an arrhythmia clinic, including a 48h Holter monitor at each follow-up point. |
| Inamura (2021)^64^ | Obs | N | Japan | COMBINED | Consecutive patients who underwent an initial catheter ablation for paroxysmal or persistent AF at the Japanese Red Cross Saitama Hospital. | PVI ± LA posterior wall isolation ± Linear ablation ± non-PV triggers ablation | 2013-2019 | 2.1 ± 1.5 | Any atrial tachyarrhythmia lasting >30 s after the 3-month blanking period. | The first outpatient clinic visit occurred 2–3 weeks after the procedure. Subsequent follow-up visits consisted of a clinical interview and ECG every 2–3 months and a 24h Holter monitoring, or a 2-week cardiac event recording at 3 and 12 months after the procedure. |
| Jastrzebski (2021)^65^ | Obs | N | Poland | CRYO | Consecutive patients with symptomatic paroxysmal, persistent and long-standing persistent AF who failed one or more AADs. | PVI | 2009-2019 | NR | The first episode of AF lasting > 30 s diagnosed after the blanking period of 3 months. AF had to be documented by ECG, Holter monitoring or physician interpreted intracardiac ECG from the implanted device (event marker data were considered unreliable). | Holter monitoring (24–72 h long) was scheduled after 3 and 6–9 months post-ablation and then advised once a year. |
| Maier (2021)^66^ | Obs | N | USA | CRYO | Patients suffering from paroxysmal or non-paroxysmal AF who underwent their first PVI by cryoballoon ablation at the Kepler University Hospital in Linz, Austria. | PVI | 2009-2017 | 4.5 ± 2.4 | (1) an instance of AF was detected on an electrocardiogram (ECG-at least 30s in duration) and/or (2) an electric cardioversion due to AF recurrence and/or (3) a re-do of the procedure due to AF recurrence were necessary.  3 months of blanking period. | Outpatient check-ups at our clinic were scheduled for patients at 3 and 12 months post-intervention. A routine Holter ECG and resting ECG was performed after 3 and 12 months, respectively. Additional resting ECGs, event recorders or 24-hrs Holter ECGs were utilised to verify AF recurrence in case of symptoms typical of AF. |
| Mugnai (2021)^67^ | Obs | Y | Belgium | CRYO | All patients referred our electrophysiology centre of UZ Brussel (Brussels, Belgium) from October 2012 were retrospectively analysed. | PVI | 2012-? | 5.1 ± 0.6 | Symptomatic and/or documented episodes of atrial tachyarrhythmias lasting more than 30s.  3 months of blanking period. | After discharge from the hospital, patients were scheduled for follow-up visits at 1, 3, 6, 12 months and biannually thereafter. A 12-lead ECG and 24-h-Holter monitoring were reviewed during the clinical evaluations; previously implanted pacemakers and cardioverter-defibrillators were interrogated during the visits. |
| Sinkovec (2021)^68^ | Obs | N | Slovenia | RFA | Consecutive patients who underwent catheter ablation of symptomatic paroxysmal or persistent AF in the period due to unsuccessful AAD treatment. | PVI ± non-PV triggers ablation ± CTI ablation | 2006-2009 | 11.1 ± 2.9 | Arrhythmia recurrence not clearly defined.  3 months of blanking period. | Patients were asked to return for a follow-up in 3–6 months, then in one year, and later in case of palpitations. |
| Wen (2021)^69^ | Obs | Y | US | RFA | Patients with symptomatic and drug-refractory AF who underwent primary AF ablation at Mayo Clinic (Rochester, MN). | PVI ± Linear ablation ± non-PV triggers ablation | 2011-2017 | 3.0 ± 0.0 | Any atrial tachyarrhythmia including documented AF, atrial flutter, or AT lasting > 30s and occurred after the 3-month blanking period. | Patients were followed by both Mayo Clinic staff and their local cardiologists by means of telephone interviews at 30 days post-ablation, office visits, at months 3 and 12, then every 12 months thereafter |
| Wu (2021)^70^ | RCT | N | China | RFA | The subjects were ≥18 and ≤75years of age, and women were not lactating or pregnant. | PVI ± Linear ablation ± CFAE ablation | 2012-2014 | 4.5 ± 0.9 | AF, atrial flutter, or AT lasting >30, as documented by ECG or a device-recording system following the 3 months post-ablation blank period. | The patients returned for a follow-up visit after the initial therapy and at 3, 6, 12, 18, 24, 30, 36, 42, 48, 54, and 60 months thereafter. During each visit, the patients’ history, physical examination, echocardiography, ECG, and Holter monitoring or 1-week event recorder were obtained. |
| Baimbetov (2022)^71^ | Obs | N | Ireland | CRYO | Consecutive patients with paroxysmal AF resistant to antiarrhythmic therapy (including cordarone) from November 2014 to November 2016. | PVI | 2014-2016 | 4.1 ± 1.5 | Documented arrhythmias lasting > 30 s or symptoms indicating arrhythmia recurrence, with or without AAD.  3 months of blanking period. | The patients were followed-up with constant ECG monitoring in the hospital for 3 days after the procedure. The first visit to the clinic was 4 weeks after the procedure. Subsequent visits consisted of a clinical interview, ECG, and a 24-h Holter monitoring at 3, 6, 9, and 12 months in the clinic, in addition to a routine follow-up by an independent physician. The patients then visited the clinic every 6 months. |
| Schlogl (2022)^72^ | Obs | N | Germany | RFA | Consecutive patients with AF undergoing catheter ablation. | PVI ± Linear ablation | 2006-2016 | 2.3 ± 2.3 | A documented AF/AT episode lasting > 30 s after the 3-month blanking period. | After hospital discharge, patients were followed in our outpatient clinic and a 4-day continuous Holter ECG was repeated after 3, 6 and 12 months and on a 12-month basis thereafter. |
| Simon (2022)^73^ | Obs | Y | Hungary | RFA | Consecutive patients with symptomatic AF who underwent initial point-by-point radiofrequency catheter ablation at the Heart and Vascular Center of Semmelweis University, Budapest, Hungary. | PVI ± Linear ablation | 2014-2017 | NR | The occurrence of atrial tachyarrhythmia that lasted >30 s with or without symptoms.  3 months of blanking period. | After discharge, outpatient clinical follow-up visits were scheduled at 3, 6, and 12 months after the procedure and at least once yearly thereafter. The follow-up visits included clinical assessment of the patient and 24-hour Holter ECG monitoring. |

Footnote: AAD = anti-arrhythmic drug, AF = atrial fibrillation, AT = atrial tachycardia, ECG = electrocardiography, PVI = pulmonary vein isolation; RFA = radiofrequency ablation; NR = not reported; NA = not applicable; Obs = observational; CRYO = cryoballoon; SVC = superior vena cava; CTI = cavotricuspid isthmus; PAF = paroxysmal atrial fibrillation; CFAE = cardiac fractionated atrial electrocardiograms.

*Linear ablation includes the roof line, posterior inferior wall, and mitral isthmus ablation.

**Table S3: Quality assessment of individual studies**

| **First author (year)** | **Type of tool used** | **Quality** | **Questions** | | | | | | | | | | | | | |  |
| --- | --- | --- | --- | --- | --- | --- | --- | --- | --- | --- | --- | --- | --- | --- | --- | --- | --- |
|  |  |  | 1 | 2 | 3 | 4 | 5 | 6 | 7 | 8 | 9 | 10 | 11 | 12 | 13 | 14 | |
| Nademanee (2008)^1^ | Case series | FAIR | Y | Y | Y | Y | Y | Y | Y | Y | N |  |  |  |  |  | |
| Bhargava (2009)^2^ | Case series | GOOD | Y | Y | Y | Y | Y | Y | Y | Y | Y |  |  |  |  |  | |
| Sawhney (2009)^3^ | Case series | GOOD | Y | Y | Y | Y | Y | Y | Y | Y | Y |  |  |  |  |  | |
| Hunter (2010)^4^ | Case series | FAIR | Y | Y | Y | Y | Y | Y | Y | N | Y |  |  |  |  |  | |
| Tzou (2010)^5^ | Case series | FAIR | Y | Y | Y | Y | N | Y | Y | N | Y |  |  |  |  |  | |
| Daly (2011)^6^ | Case series | FAIR | Y | Y | Y | Y | Y | N | Y | Y | Y |  |  |  |  |  | |
| Fiala (2012)^7^ | Case series | FAIR | Y | Y | Y | Y | N | Y | Y | Y | Y |  |  |  |  |  | |
| Hunter (2012)^8^ | Case series | FAIR | Y | N | Y | Y | Y | Y | Y | Y | Y |  |  |  |  |  | |
| Sorgente (2012)^9^ | Case series | FAIR | Y | Y | Y | Y | Y | Y | Y | N | Y |  |  |  |  |  | |
| Neumann (2013)^10^ | Case series | GOOD | Y | Y | Y | Y | Y | Y | Y | Y | Y |  |  |  |  |  | |
| Uchiyama (2013)^11^ | Case series | FAIR | Y | Y | N | Y | Y | Y | Y | N | Y |  |  |  |  |  | |
| Wang (2013)^12^ | Case series | GOOD | Y | Y | Y | Y | Y | Y | Y | Y | Y |  |  |  |  |  | |
| Wojcik (2013)^13^ | Case series | GOOD | Y | Y | Y | Y | Y | Y | Y | Y | Y |  |  |  |  |  | |
| Zhou (2013)^14^ | Case series | FAIR | Y | Y | Y | Y | Y | Y | Y | N | Y |  |  |  |  |  | |
| Gaita (2014)^15^ | Case series | GOOD | Y | Y | Y | Y | Y | Y | Y | Y | Y |  |  |  |  |  | |
| Gal (2014)^16^ | Controlled Intervention Studies | POOR | Y | N | NR | NR | NR | Y | Y | Y | Y | Y | Y | N | N | Y | |
| Hayashi (2014)^17^ | Case series | FAIR | Y | Y | Y | Y | Y | Y | Y | N | Y |  |  |  |  |  | |
| Takigawa (2014)^18^ | Case series | GOOD | Y | Y | Y | Y | Y | Y | Y | Y | Y |  |  |  |  |  | |
| Costa (2015)^19^ | Case series | FAIR | Y | Y | Y | N | Y | Y | Y | Y | Y |  |  |  |  |  | |
| Karasoy (2015)^20^ | Case series | GOOD | Y | Y | Y | Y | Y | Y | Y | Y | Y |  |  |  |  |  | |
| Noseworthy (2015)^21^ | Case series | GOOD | Y | Y | Y | Y | NA | Y | Y | Y | Y |  |  |  |  |  | |
| Schreiber (2015)^22^ | Case series | GOOD | Y | Y | Y | Y | Y | Y | Y | Y | Y |  |  |  |  |  | |
| Tran (2015)^23^ | Case series | POOR | Y | Y | N | N | Y | N | Y | Y | Y |  |  |  |  |  | |
| Yamaguchi (2015)^24^ | Case series | GOOD | Y | Y | Y | Y | Y | Y | Y | Y | Y |  |  |  |  |  | |
| Bunch (2016)^25^ | Case series | FAIR | Y | Y | Y | Y | N | Y | Y | Y | Y |  |  |  |  |  | |
| Teunissen (2016)^26^ | Case series | FAIR | Y | Y | N | Y | Y | Y | Y | Y | N |  |  |  |  |  | |
| Hung (2017)^27^ | Case series | FAIR | Y | Y | Y | Y | Y | Y | N | N | Y |  |  |  |  |  | |
| Kawaji (2017)^28^ | Case series | GOOD | Y | Y | Y | Y | Y | Y | Y | Y | Y |  |  |  |  |  | |
| Miyazaki (2016)^29^ | Case series | GOOD | Y | Y | Y | Y | Y | Y | Y | Y | Y |  |  |  |  |  | |
| Nielsen (2017)^30^ | Controlled Intervention Studies | POOR | Y | Y | N | N | N | Y | Y | Y | Y |  |  |  |  |  | |
| Saliba (2017)^31^ | Case series | FAIR | Y | Y | Y | Y | NA | N | Y | Y | N |  |  |  |  |  | |
| Winkle (2017)^32^ | Case series | FAIR | Y | Y | Y | Y | Y | Y | Y | N | N |  |  |  |  |  | |
| Yagishita (2017)^33^ | Case series | GOOD | Y | Y | Y | Y | Y | Y | Y | Y | Y |  |  |  |  |  | |
| Yin (2017)^34^ | Case series | GOOD | Y | Y | Y | Y | Y | Y | Y | Y | Y |  |  |  |  |  | |
| Akkaya (2018)^35^ | Case series | GOOD | Y | Y | Y | Y | Y | Y | Y | Y | Y |  |  |  |  |  | |
| Ang (2018)^36^ | Case series | POOR | Y | NR | N | N | N | Y | Y | Y | Y | Y | Y | N | Y | Y | |
| Chelu (2018)^37^ | Case series | FAIR | N | Y | Y | Y | Y | Y | Y | Y | Y |  |  |  |  |  | |
| De Greef (2018)^38^ | Case series | FAIR | N | Y | Y | Y | Y | Y | Y | N | Y |  |  |  |  |  | |
| De Maat (2018)^39^ | Case series | FAIR | N | Y | Y | Y | Y | Y | Y | Y | Y |  |  |  |  |  | |
| Fredersdorf (2018)^40^ | Case series | FAIR | Y | Y | Y | Y | Y | Y | Y | N | Y |  |  |  |  |  | |
| Gaita (2018)^41^ | Case series | FAIR | N | Y | Y | Y | Y | N | Y | Y | Y |  |  |  |  |  | |
| Kim (2018)^42^ | Case series | POOR | Y | Y | Y | Y | N | Y | Y | N | N |  |  |  |  |  | |
| Lee (2018)^43^ | Controlled Intervention study | FAIR | Y | Y | Y | Y | NR | Y | Y | Y | Y | Y | Y | Y | Y | Y | |
| Reissmann (2018)^44^ | Case series | GOOD | Y | Y | Y | Y | Y | Y | Y | Y | Y |  |  |  |  |  | |
| Tilz (2018)^45^ | Case series | GOOD | Y | Y | Y | Y | Y | Y | Y | Y | Y |  |  |  |  |  | |
| Canpolat (2019)^46^ | Case series | FAIR | Y | Y | Y | Y | Y | Y | Y | N | Y |  |  |  |  |  | |
| Efremidis (2019)^47^ | Case series | FAIR | N | Y | Y | Y | Y | Y | Y | N | Y |  |  |  |  |  | |
| Gedikli (2019)^48^ | Case series | GOOD | Y | Y | Y | Y | Y | Y | Y | Y | Y |  |  |  |  |  | |
| Kim (2019)^49^ | Case series | FAIR | Y | Y | Y | N | Y | Y | Y | N | Y |  |  |  |  |  | |
| Kornej (2019)^50^ | Case series | FAIR | Y | Y | Y | Y | Y | Y | Y | N | Y |  |  |  |  |  | |
| Lin (2019)^51^ | Case series | FAIR | Y | Y | Y | Y | NA | N | Y | Y | Y |  |  |  |  |  | |
| Packer (2019)^52^ | Controlled Intervention study | GOOD | Y | Y | Y | Y | Y | Y | Y | Y | Y |  |  |  |  |  | |
| Baba (2020)^53^ | Case series | POOR | Y | N | Y | Y | Y | Y | Y | N | N |  |  |  |  |  | |
| Baek (2020)^54^ | Case series | FAIR | N | Y | Y | Y | Y | Y | Y | N | Y |  |  |  |  |  | |
| Heeger (2020)^55^ | Case series | FAIR | N | Y | Y | Y | Y | Y | Y | N | Y |  |  |  |  |  | |
| Kis (2020)^56^ | Case series | FAIR | Y | Y | Y | Y | N | N | Y | Y | Y |  |  |  |  |  | |
| Kriatselis (2020)^57^ | Case series | FAIR | Y | Y | Y | Y | Y | Y | Y | N | Y |  |  |  |  |  | |
| Romero (2020)^58^ | Case series | FAIR | N | Y | Y | N | Y | Y | Y | Y | Y |  |  |  |  |  | |
| Sawhney (2020)^59^ | Case series | FAIR | Y | Y | Y | Y | Y | Y | Y | N | Y |  |  |  |  |  | |
| Sugumar (2020)^60^ | Case series | FAIR | N | Y | Y | Y | Y | Y | Y | Y | Y |  |  |  |  |  | |
| Ding (2021)^61^ | Case series | FAIR | N | Y | Y | Y | Y | Y | Y | Y | Y |  |  |  |  |  | |
| Esato (2021)^62^ | Case series | GOOD | Y | Y | Y | Y | Y | Y | Y | Y | Y |  |  |  |  |  | |
| Gallagher (2021)^63^ | Controlled Intervention Studies | POOR | Y | NR | NR | NR | NR | Y | Y | Y | Y | Y | Y | Y | N | Y | |
| Inamura (2021)^64^ | Case series | FAIR | N | Y | Y | Y | Y | Y | Y | Y | Y |  |  |  |  |  | |
| Jastrzebski (2021)^65^ | Case series | GOOD | Y | Y | Y | Y | Y | Y | Y | Y | Y |  |  |  |  |  | |
| Maier (2021)^66^ | Case series | FAIR | N | Y | Y | Y | Y | Y | Y | Y | Y |  |  |  |  |  | |
| Mugnai (2021)^67^ | Case series | FAIR | Y | Y | Y | Y | N | Y | Y | Y | N |  |  |  |  |  | |
| Sinkovec (2021)^68^ | Case series | FAIR | N | Y | Y | Y | Y | Y | Y | Y | Y |  |  |  |  |  | |
| Wen (2021)^69^ | Case series | GOOD | Y | Y | Y | Y | Y | Y | Y | Y | Y |  |  |  |  |  | |
| Wu (2021)^70^ | Controlled Intervention Studies | POOR | Y | Y | N | N | NR | Y | Y | Y | Y | Y | Y | Y | NA | Y | |
| Baimbetov (2022)^71^ | Case series | FAIR | N | Y | Y | Y | Y | Y | Y | Y | Y |  |  |  |  |  | |
| Schlogl (2022)^72^ | Case series | GOOD | Y | Y | Y | Y | Y | Y | Y | Y | Y |  |  |  |  |  | |
| Simon (2022)^73^ | Case series | GOOD | Y | Y | Y | Y | Y | Y | Y | Y | Y |  |  |  |  |  | |

Footnote: RCT = randomized controlled trial, N = No, NA = not applicable, NR = not reported, Y = Yes.

**Table S4: Comparison of results with arcsine and logit transformation**

| **Type of transformation**  **Outcome** | **Arcsine** | **Logit** |
| --- | --- | --- |
|  | **Pooled estimate (95%CI)** | **Pooled estimate (95%CI)** |
| **Freedom from atrial arrhythmia recurrence** | | |
| After single procedure | 50.6% (45.5% - 55.7%) | 50.7% (45.2% - 56.1%) |
| After multiple procedures | 69.7% (63.8% - 75.3%) | 71.1% (64.8% - 76.7%) |
| Patients with PAF, after single procedure | 59.0% (49.6% - 68.1%) | 59.7% (48.9% - 69.6%) |
| Patients with PAF, after multiple procedures | 80.8% (73.9% - 86.9%) | 81.5% (73.9% - 87.3%) |
| Patients with non-PAF, after single procedure | 34.1% (22.8% - 46.4%) | 33.3% (22.4% - 46.4%) |
| Patients with non-PAF, after multiple procedures | 60.6% (50.6% - 70.2%) | 60.9% (50.4% - 70.5%) |
| Prospective studies, after single procedure | 48.7% (42.0% - 55.4%) | 48.6% (41.6% - 55.6%) |
| Prospective studies, after multiple procedures | 69.3% (61.0% - 77.1%) | 70.1% (61.8% - 78.7%) |
| Retrospective studies, after single procedure | 54.1% (46.7% - 61.4%) | 54.6% (46.5% - 62.4%) |
| Retrospective studies, after multiple procedures | 71.4% (63.1% - 79.0%) | 72.5% (63.7% - 79.8%) |
| Good studies, after single procedure | 44.9% (36.6% - 53.2%) | 44.4% (35.9% - 53.3%) |
| Good studies, after multiple procedures | 71.8% (62.5% - 80.2%) | 72.7% (63.1% - 80.6%) |
| Fair studies, after single procedure | 53.7% (48.1% - 59.3%) | 53.9% (48.0% - 59.7%) |
| Fair studies, after multiple procedures | 66.6% (59.0% - 73.8%) | 67.6% (59.4% - 74.9%) |
| Poor studies, after single procedure | 53.3% (24.1% - 81.2%) | 55.3% (22.5% - 84.1%) |
| Poor studies, after multiple procedures | 76.6% (53.6% - 93.5%) | 80.4% (53.1% - 93.7%) |
| RF, after single procedure | 49.1% (42.6% - 55.6%) | 49.1% (42.1% - 56.2%) |
| RF, after multiple procedures | 68.0% (61.7% - 74.0%) | 69.1% (62.3% - 75.1%) |
| CRYO, after single procedure | 56.9% (51.2% - 62.5%) | 57.0% (51.1% - 62.7%) |
| CRYO, after multiple procedures | 75.7% (61.6% - 87.4%) | 76.5% (59.4% - 87.9%) |
| **Other clinical outcomes at 5-years following AF ablation** | | |
| Mortality | 6.0% (3.2% - 9.7%) | 5.6% (3.1% - 9.9%) |
| Stroke | 2.4% (1.4% - 3.7%) | 2.3% (1.4% - 3.7%) |
| Bleeding | 1.2% (0.8% - 2.0%) | 1.3% (0.8% - 2.0%) |

Footnote: AF=atrial fibrillation, RF=radiofrequency, CRYO=cryoablation, NA = not applicable due to insufficient data.

**Figure S1: Funnel plots of the meta-analysis of freedom from atrial arrhythmia recurrence after single procedure**


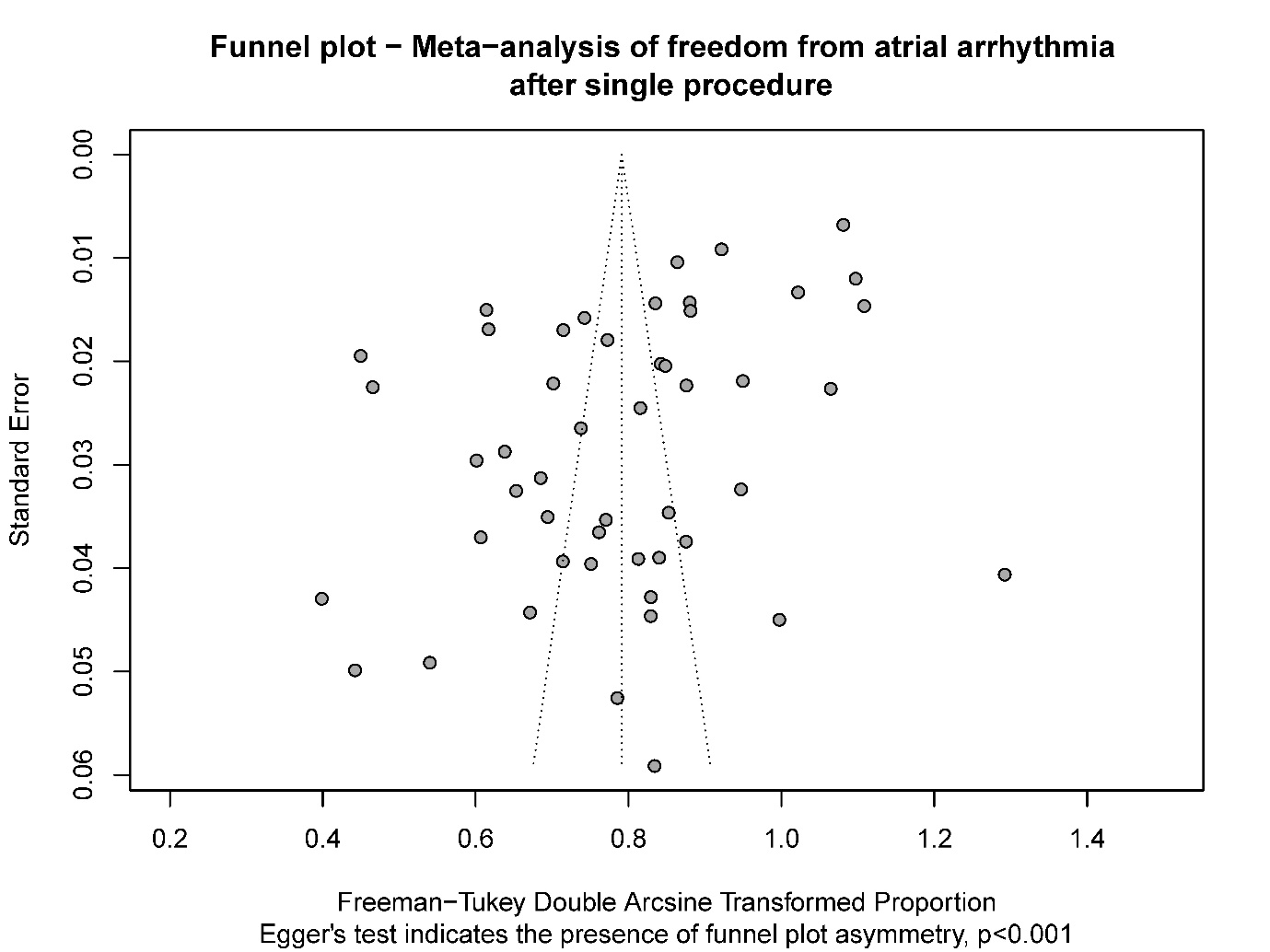


**Figure S2: Funnel plots of the meta-analysis of freedom from atrial arrhythmia recurrence after multiple procedures**

**
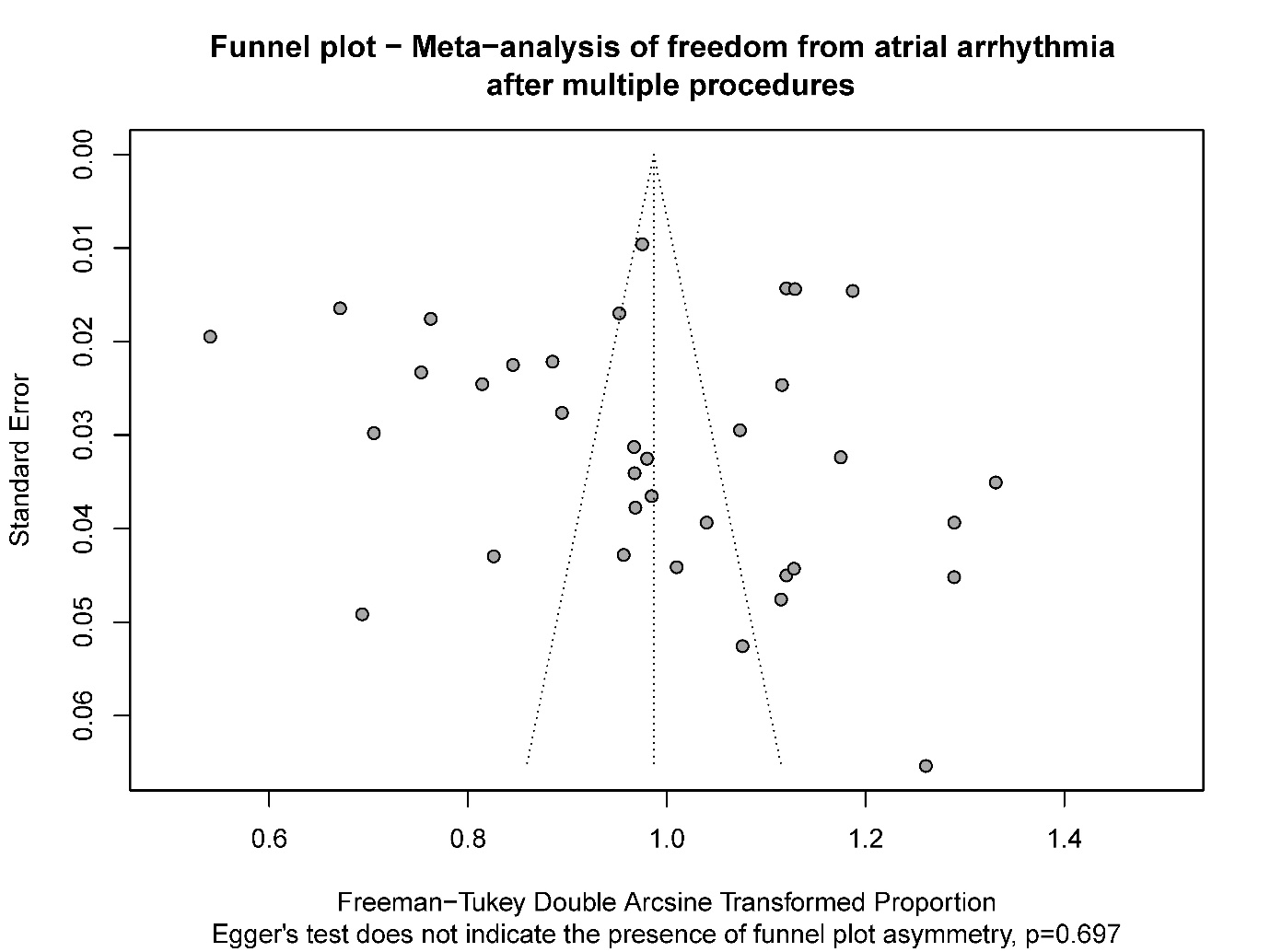
**

**Figure S3: Funnel plots of the meta-analysis of all-cause mortality**


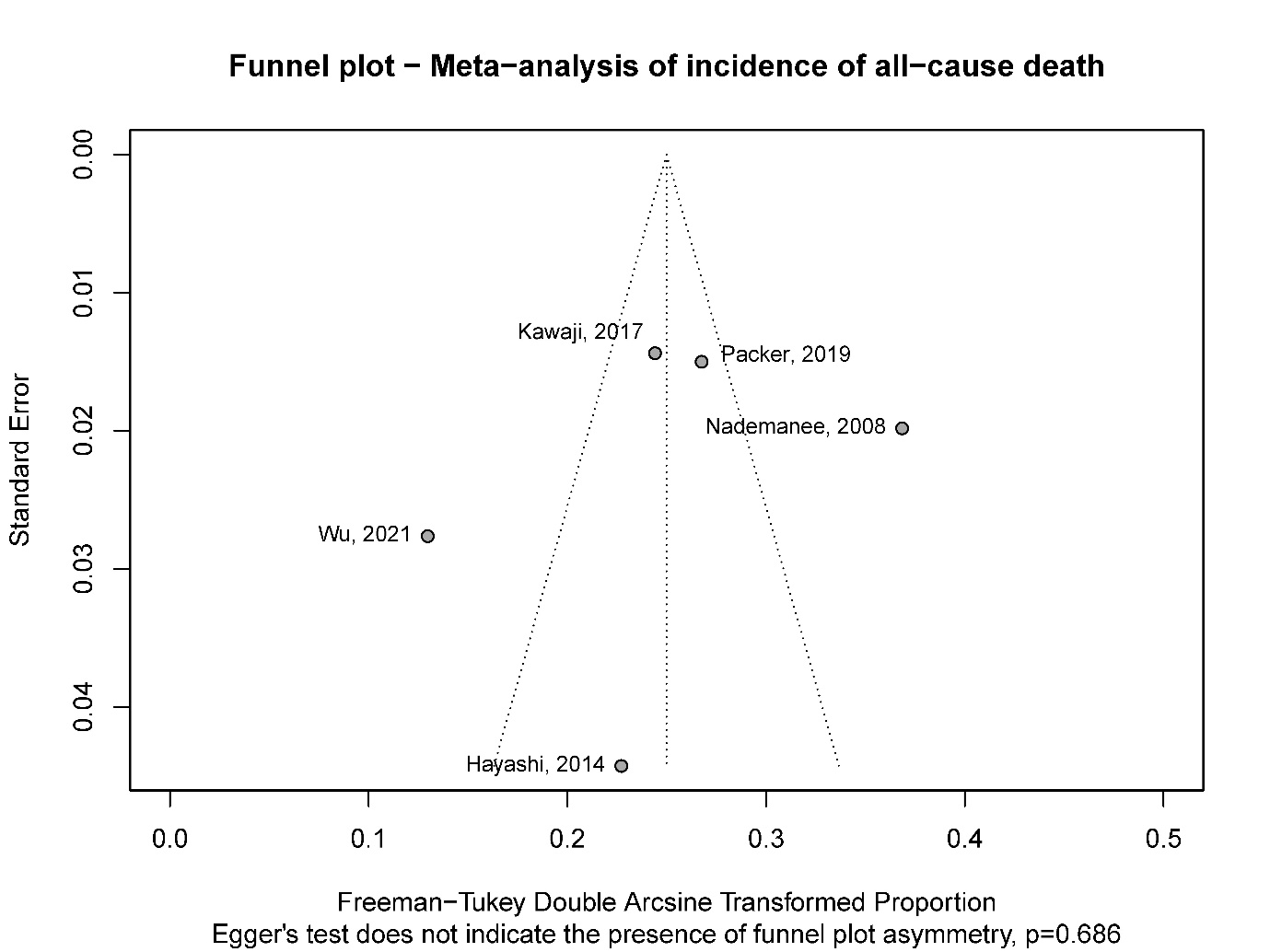


**Figure S4: Funnel plots of the meta-analysis of stroke or transient ischaemic attack**


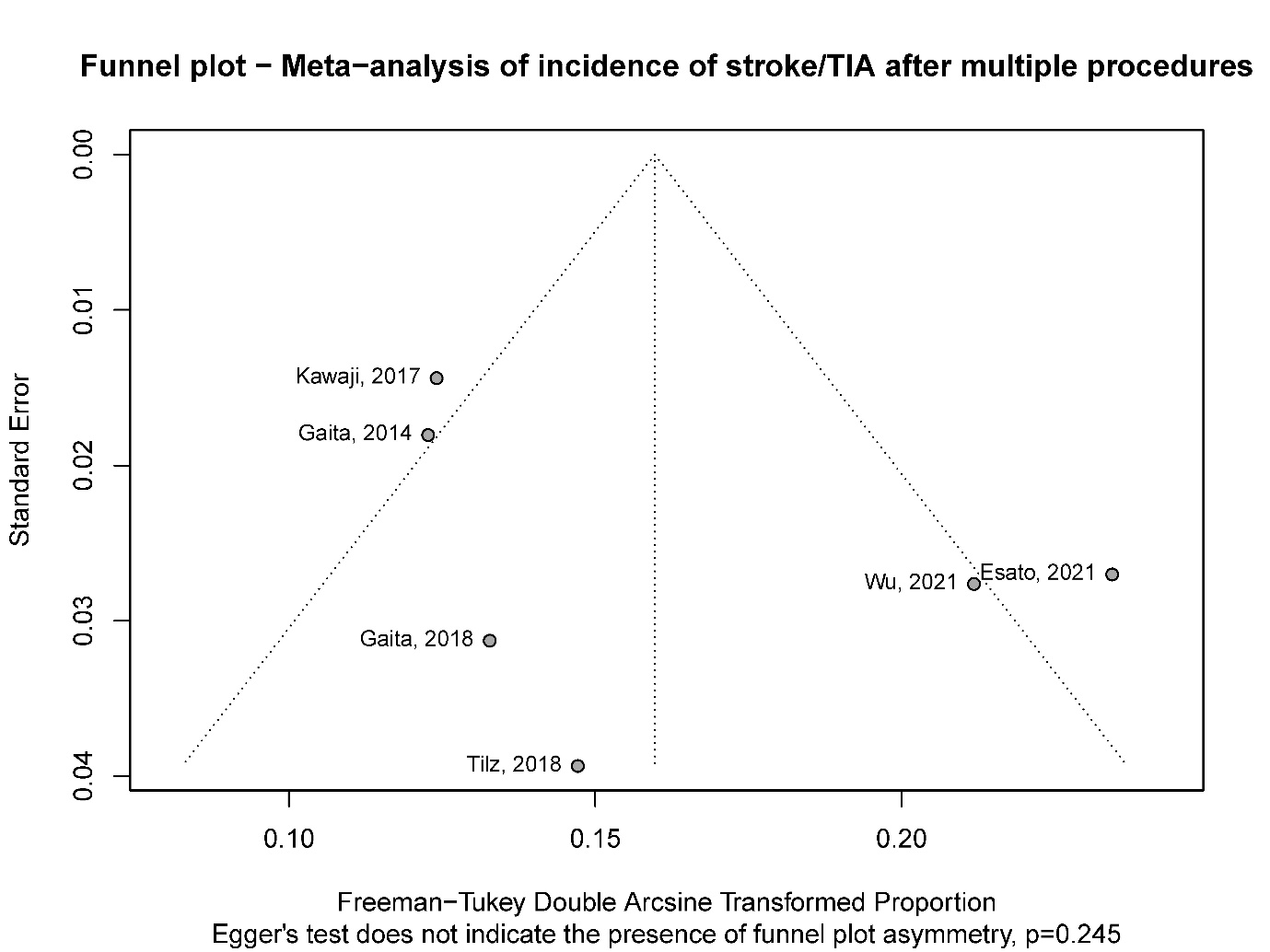


**Figure S5: Funnel plots of the meta-analysis of major bleeding**


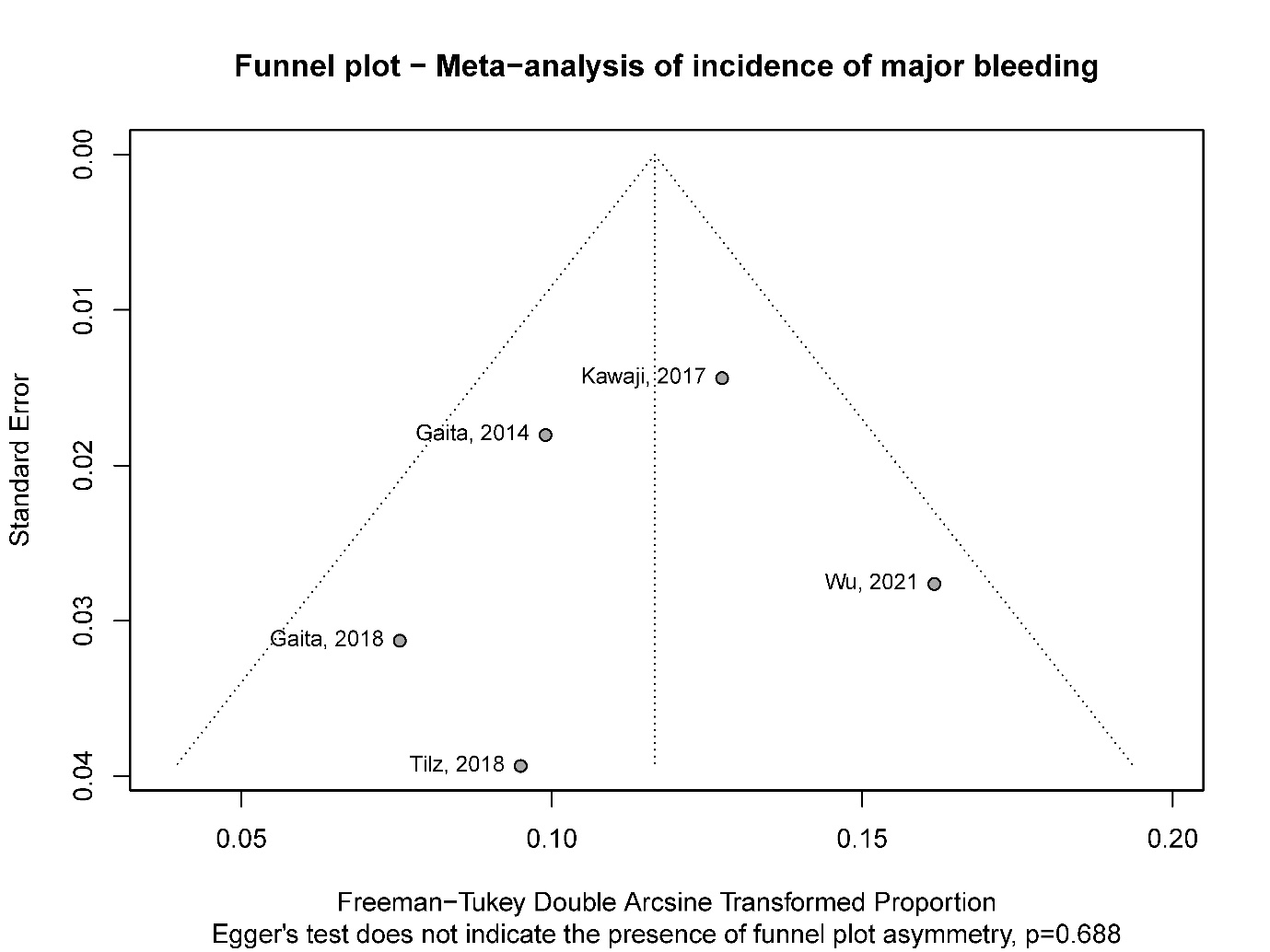


**Figure S6: Leave-one-out analysis for the meta-analysis of freedom from atrial arrhythmia recurrence at 5-years after a single procedure**

**
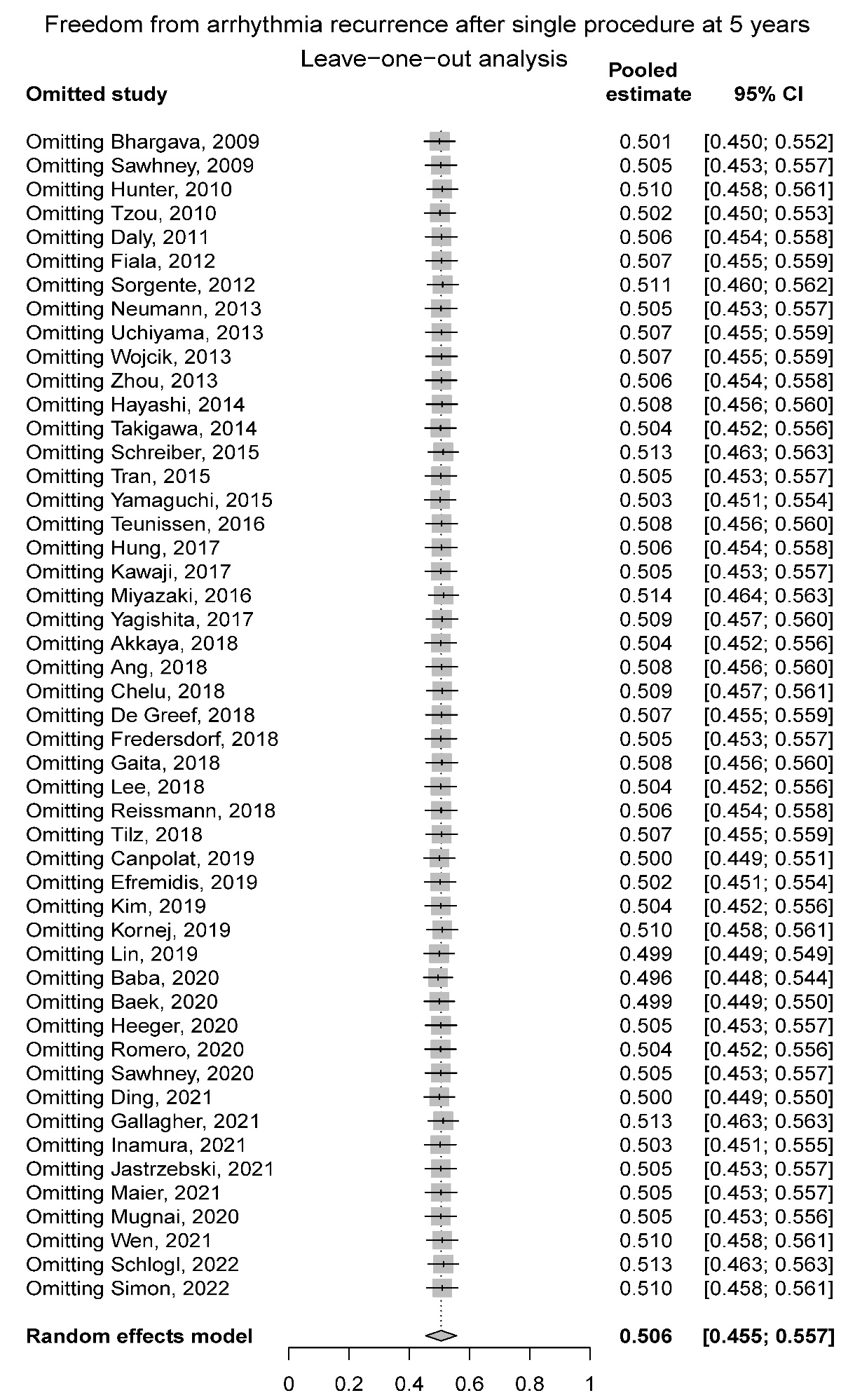
**

**Figure S7: Bias-corrected trim-and-fill funnel plot for meta-analysis of freedom from atrial arrhythmia recurrence at 5-years after a single procedure**


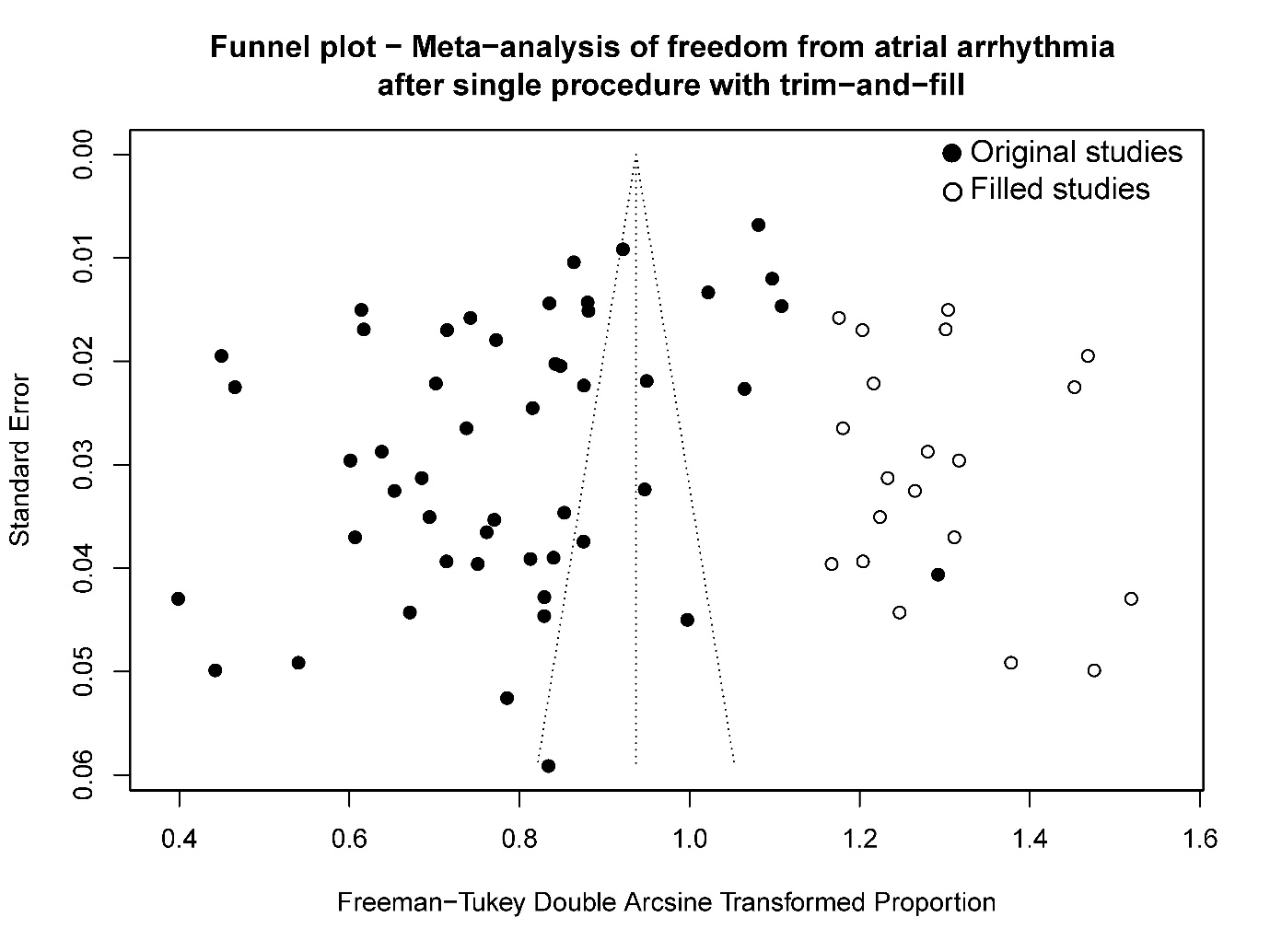


**References**

1. Nademanee K, Schwab MC, Kosar EM, et al. Clinical outcomes of catheter substrate ablation for high-risk patients with atrial fibrillation. J Am Coll Cardiol 2008;51(8):843-9. (In eng). DOI: 10.1016/j.jacc.2007.10.044.

2. Bhargava M, Di Biase L, Mohanty P, et al. Impact of type of atrial fibrillation and repeat catheter ablation on long-term freedom from atrial fibrillation: results from a multicenter study. Heart Rhythm 2009;6(10):1403-12. (In eng). DOI: 10.1016/j.hrthm.2009.06.014.

3. Sawhney N, Anousheh R, Chen WC, Narayan S, Feld GK. Five-year outcomes after segmental pulmonary vein isolation for paroxysmal atrial fibrillation. Am J Cardiol 2009;104(3):366-72. (In eng). DOI: 10.1016/j.amjcard.2009.03.044.

4. Hunter RJ, Berriman TJ, Diab I, et al. Long-term efficacy of catheter ablation for atrial fibrillation: impact of additional targeting of fractionated electrograms. Heart 2010;96(17):1372-8. (In eng). DOI: 10.1136/hrt.2009.188128.

5. Tzou WS, Marchlinski FE, Zado ES, et al. Long-Term Outcome After Successful Catheter Ablation of Atrial Fibrillation. Circulation: Arrhythmia and Electrophysiology 2010;3(3):237-242. DOI: 10.1161/CIRCEP.109.923771.

6. Daly M, Melton I, Crozier I. Pulmonary vein ablation for atrial fibrillation: The Christchurch, New Zealand experience. New Zealand Medical Journal 2011;124(1343):39-47. (Article) (In English) (<http://www.embase.com/search/results?subaction=viewrecord&id=L362627676&from=exporthttp://journal.nzma.org.nz/journal/124-1343/4878/content.pdf>).

7. Fiala M, Škňouřil L, Toman O, et al. Long-term results of catheter ablation for atrial fibrillation in 866 patients. Cor et Vasa 2012;54(6):e361-e368. (Article) (In English). DOI: 10.1016/j.crvasa.2012.10.006.

8. Hunter RJ, McCready J, Diab I, et al. Maintenance of sinus rhythm with an ablation strategy in patients with atrial fibrillation is associated with a lower risk of stroke and death. Heart 2012;98(1):48-53. (In eng). DOI: 10.1136/heartjnl-2011-300720.

9. Sorgente A, Tung P, Wylie J, Josephson ME. Six year follow-up after catheter ablation of atrial fibrillation: a palliation more than a true cure. Am J Cardiol 2012;109(8):1179-86. DOI: 10.1016/j.amjcard.2011.11.058.

10. Neumann T, Wojcik M, Berkowitsch A, et al. Cryoballoon ablation of paroxysmal atrial fibrillation: 5-year outcome after single procedure and predictors of success. Europace 2013;15(8):1143-9. (In eng). DOI: 10.1093/europace/eut021.

11. Uchiyama T, Miyazaki S, Taniguchi H, et al. Six-year follow-up of catheter ablation in paroxysmal atrial fibrillation. Circ J 2013;77(11):2722-7. (In eng). DOI: 10.1253/circj.cj-13-0468.

12. Wang K, Chang D, Chu Z, et al. Denervation as a common mechanism underlying different pulmonary vein isolation strategies for paroxysmal atrial fibrillation: evidenced by heart rate variability after ablation. ScientificWorldJournal 2013;2013:569564. (In eng). DOI: 10.1155/2013/569564.

13. Wojcik M, Erkapic D, Berkowitsch A, et al. Ipsilateral circumferential radiofrequency ablation of atrial fibrillation with irrigated tip catheter: long-term outcome and pre-procedural predictors. Circ J 2013;77(9):2280-7. (In eng). DOI: 10.1253/circj.cj-13-0275.

14. Zhou G, Chen S, Chen G, et al. Procedural arrhythmia termination and long-term single-procedure clinical outcome in patients with non-paroxysmal atrial fibrillation. J Cardiovasc Electrophysiol 2013;24(10):1092-100. (In eng). DOI: 10.1111/jce.12193.

15. Gaita F, Sardi D, Battaglia A, et al. Incidence of cerebral thromboembolic events during long-term follow-up in patients treated with transcatheter ablation for atrial fibrillation. Europace 2014;16(7):980-6. (Article) (In English). DOI: 10.1093/europace/eut406.

16. Gal P, Aarntzen AE, Smit JJ, et al. Conventional radiofrequency catheter ablation compared to multi-electrode ablation for atrial fibrillation. Int J Cardiol 2014;176(3):891-5. (Article in Press) (In English). DOI: 10.1016/j.ijcard.2014.08.034.

17. Hayashi M, Kaneko S, Shimano M, et al. Efficacy and safety of radiofrequency catheter ablation for atrial fibrillation in chronic hemodialysis patients. Nephrol Dial Transplant 2014;29(1):160-7. (In eng). DOI: 10.1093/ndt/gft233.

18. Takigawa M, Takahashi A, Kuwahara T, et al. Long-term follow-up after catheter ablation of paroxysmal atrial fibrillation: the incidence of recurrence and progression of atrial fibrillation. Circ Arrhythm Electrophysiol 2014;7(2):267-73. (In eng). DOI: 10.1161/CIRCEP.113.000471.

19. Costa FM, Ferreira AM, Oliveira S, et al. Left atrial volume is more important than the type of atrial fibrillation in predicting the long-term success of catheter ablation. Int J Cardiol 2015;184:56-61. (In eng). DOI: 10.1016/j.ijcard.2015.01.060.

20. Karasoy D, Gislason GH, Hansen J, et al. Oral anticoagulation therapy after radiofrequency ablation of atrial fibrillation and the risk of thromboembolism and serious bleeding: long-term follow-up in nationwide cohort of Denmark. Eur Heart J 2015;36(5):307-14a. (In eng). DOI: 10.1093/eurheartj/ehu421.

21. Noseworthy PA, Kapa S, Deshmukh AJ, et al. Risk of stroke after catheter ablation versus cardioversion for atrial fibrillation: A propensity-matched study of 24,244 patients. Heart Rhythm 2015;12(6):1154-61. (In eng). DOI: 10.1016/j.hrthm.2015.02.020.

22. Schreiber D, Rostock T, Frohlich M, et al. Five-year follow-up after catheter ablation of persistent atrial fibrillation using the stepwise approach and prognostic factors for success. Circ Arrhythm Electrophysiol 2015;8(2):308-17. (In eng). DOI: 10.1161/CIRCEP.114.001672.

23. Tran VN, Tessitore E, Gentil-Baron P, et al. Thromboembolic events 7-11 years after catheter ablation of atrial fibrillation. Pacing Clin Electrophysiol 2015;38(4):499-506. (In eng). DOI: 10.1111/pace.12588.

24. Yamaguchi Y, Sohara H, Takeda H, et al. Long-Term Results of Radiofrequency Hot Balloon Ablation in Patients With Paroxysmal Atrial Fibrillation: Safety and Rhythm Outcomes. J Cardiovasc Electrophysiol 2015;26(12):1298-306. (In eng). DOI: 10.1111/jce.12820.

25. Bunch TJ, May HT, Bair TL, et al. The Impact of Age on 5-Year Outcomes After Atrial Fibrillation Catheter Ablation. J Cardiovasc Electrophysiol 2016;27(2):141-6. (In eng). DOI: 10.1111/jce.12849.

26. Teunissen C, Kassenberg W, van der Heijden JF, et al. Five-year efficacy of pulmonary vein antrum isolation as a primary ablation strategy for atrial fibrillation: a single-centre cohort study. Europace 2016;18(9):1335-42. (Article) (In English). DOI: 10.1093/europace/euv439.

27. Hung Y, Lo LW, Lin YJ, et al. Characteristics and long-term catheter ablation outcome in long-standing persistent atrial fibrillation patients with non-pulmonary vein triggers. Int J Cardiol 2017;241:205-211. (In eng). DOI: 10.1016/j.ijcard.2017.04.050.

28. Kawaji T, Shizuta S, Morimoto T, et al. Very long-term clinical outcomes after radiofrequency catheter ablation for atrial fibrillation: A large single-center experience. Int J Cardiol 2017;249:204-213. (In eng). DOI: 10.1016/j.ijcard.2017.09.023.

29. Miyazaki S, Taniguchi H, Kusa S, et al. Five-year follow-up outcome after catheter ablation of persistent atrial fibrillation using a sequential biatrial linear defragmentation approach: What does atrial fibrillation termination during the procedure imply? Heart Rhythm 2017;14(1):34-40. (In eng). DOI: 10.1016/j.hrthm.2016.08.041.

30. Nielsen JC, Johannessen A, Raatikainen P, et al. Long-term efficacy of catheter ablation as first-line therapy for paroxysmal atrial fibrillation: 5-year outcome in a randomised clinical trial. Heart 2017;103(5):368-376. (In eng). DOI: 10.1136/heartjnl-2016-309781.

31. Saliba W, Schliamser JE, Lavi I, Barnett-Griness O, Gronich N, Rennert G. Catheter ablation of atrial fibrillation is associated with reduced risk of stroke and mortality: A propensity score–matched analysis. Heart Rhythm 2017;14(5):635-642. (Article) (In English). DOI: 10.1016/j.hrthm.2017.02.001.

32. Winkle RA, Mead RH, Engel G, et al. Impact of obesity on atrial fibrillation ablation: Patient characteristics, long-term outcomes, and complications. Heart Rhythm 2017;14(6):819-827. (In eng). DOI: 10.1016/j.hrthm.2017.02.023.

33. Yagishita A, Yamauchi Y, Sato H, et al. Efficacy of Catheter Ablation and Concomitant Antiarrhythmic Drugs on the Reduction of the Arrhythmia Burden in Patients with Long-Standing Persistent Atrial Fibrillation. J Atr Fibrillation 2017;10(3):1649. (Article) (In English). DOI: 10.4022/jafib.1649.

34. Yin X, Zhao Z, Gao L, et al. Frequency Gradient Within Coronary Sinus Predicts the Long-Term Outcome of Persistent Atrial Fibrillation Catheter Ablation. J Am Heart Assoc 2017;6(3) (In eng). DOI: 10.1161/jaha.116.004869.

35. Akkaya E, Berkowitsch A, Zaltsberg S, et al. Five-year outcome and predictors of success after second-generation cryoballoon ablation for treatment of symptomatic atrial fibrillation. Int J Cardiol 2018;266:106-111. (In eng). DOI: 10.1016/j.ijcard.2018.03.069.

36. Ang R, Hunter RJ, Lim WY, et al. Long term outcome and pulmonary vein reconnection of patients undergoing cryoablation and/or radiofrequency ablation: Results from the cryo versus RF Trial. Journal of Atrial Fibrillation 2018;11(3) (Article) (In English) (<http://www.embase.com/search/results?subaction=viewrecord&id=L626172542&from=export>).

37. Chelu MG, King JB, Kholmovski EG, et al. Atrial Fibrosis by Late Gadolinium Enhancement Magnetic Resonance Imaging and Catheter Ablation of Atrial Fibrillation: 5-Year Follow-Up Data. J Am Heart Assoc 2018;7(23):e006313. (In eng). DOI: 10.1161/JAHA.117.006313.

38. De Greef Y, Schwagten B, Chierchia GB, de Asmundis C, Stockman D, Buysschaert I. Diagnosis-to-ablation time as a predictor of success: early choice for pulmonary vein isolation and long-term outcome in atrial fibrillation: results from the Middelheim-PVI Registry. Europace 2018;20(4):589-595. (In eng). DOI: 10.1093/europace/euw426.

39. De Maat GE, Mulder BA, Berretty WL, et al. Obesity is associated with impaired long-term success of pulmonary vein isolation: a plea for risk factor management before ablation. Open Heart 2018;5(1):e000771. (In eng). DOI: 10.1136/openhrt-2017-000771.

40. Fredersdorf S, Fenzl C, Jungbauer C, et al. Long-term outcomes and predictors of recurrence after pulmonary vein isolation with multielectrode ablation catheter in patients with atrial fibrillation. Journal of cardiovascular medicine (Hagerstown, Md) 2018;19(4):148-154. (In eng). DOI: 10.2459/jcm.0000000000000631.

41. Gaita F, Scaglione M, Battaglia A, et al. Very long-term outcome following transcatheter ablation of atrial fibrillation. Are results maintained after 10 years of follow up? Europace 2018;20(3):443-450. (In eng). DOI: 10.1093/europace/eux008.

42. Kim DH, Lee DI, Ahn J, et al. Ischemic stroke risk during long-term follow up in patients with successful catheter ablation for atrial fibrillation in Korea. PloS one 2018;13(7):e0201061. (In eng). DOI: 10.1371/journal.pone.0201061.

43. Lee KN, Roh SY, Baek YS, et al. Long-Term Clinical Comparison of Procedural End Points After Pulmonary Vein Isolation in Paroxysmal Atrial Fibrillation: Elimination of Nonpulmonary Vein Triggers Versus Noninducibility. Circ Arrhythm Electrophysiol 2018;11(2):e005019. (In eng). DOI: 10.1161/circep.117.005019.

44. Reissmann B, Budelmann T, Wissner E, et al. Five-year clinical outcomes of visually guided laser balloon pulmonary vein isolation for the treatment of paroxysmal atrial fibrillation. Clin Res Cardiol 2018;107(5):405-412. (In eng). DOI: 10.1007/s00392-017-1199-6.

45. Tilz RR, Heeger CH, Wick A, et al. Ten-Year Clinical Outcome After Circumferential Pulmonary Vein Isolation Utilizing the Hamburg Approach in Patients With Symptomatic Drug-Refractory Paroxysmal Atrial Fibrillation. Circ Arrhythm Electrophysiol 2018;11(2):e005250. (In eng). DOI: 10.1161/CIRCEP.117.005250.

46. Canpolat U, Kocyigit D, Yalcin MU, et al. Long-term outcomes of pulmonary vein isolation using second-generation cryoballoon during atrial fibrillation ablation. Pacing Clin Electrophysiol 2019;42(7):910-921. (In eng). DOI: 10.1111/pace.13721.

47. Efremidis M, Letsas KP, Georgopoulos S, et al. Safety, long-term outcomes and predictors of recurrence following a single catheter ablation procedure for atrial fibrillation. Acta Cardiol 2019;74(4):319-324. (In eng). DOI: 10.1080/00015385.2018.1494114.

48. Gedikli O, Mohanty S, Trivedi C, et al. Impact of dense "smoke" detected on transesophageal echocardiography on stroke risk in patients with atrial fibrillation undergoing catheter ablation. Heart Rhythm 2019;16(3):351-357. (In eng). DOI: 10.1016/j.hrthm.2018.10.004.

49. Kim JO, Shim J, Lee SH, et al. Clinical characteristics and rhythm outcome of catheter ablation of hemodynamically corrected valvular atrial fibrillation. Journal of cardiology 2019;73(6):488-496. (In eng). DOI: 10.1016/j.jjcc.2018.10.014.

50. Kornej J, Schumacher K, Zeynalova S, et al. Time-dependent prediction of arrhythmia recurrences during long-term follow-up in patients undergoing catheter ablation of atrial fibrillation: The Leipzig Heart Center AF Ablation Registry. Scientific reports 2019;9(1):7112. (Article) (In English). DOI: 10.1038/s41598-019-43644-2.

51. Lin Y, Wu HK, Wang TH, Chen TH, Lin YS. Trend and risk factors of recurrence and complications after arrhythmias radiofrequency catheter ablation: a nation-wide observational study in Taiwan. BMJ open 2019;9(5):e023487. (In eng). DOI: 10.1136/bmjopen-2018-023487.

52. Packer DL, Mark DB, Robb RA, et al. Effect of Catheter Ablation vs Antiarrhythmic Drug Therapy on Mortality, Stroke, Bleeding, and Cardiac Arrest Among Patients With Atrial Fibrillation: The CABANA Randomized Clinical Trial. Jama 2019;321(13):1261-1274. (In eng). DOI: 10.1001/jama.2019.0693.

53. Baba M, Yoshida K, Naruse Y, et al. Predictors of Recurrence after Catheter Ablation of Paroxysmal Atrial Fibrillation in Different Follow-Up Periods. Medicina (Kaunas) 2020;56(9) (In eng). DOI: 10.3390/medicina56090465.

54. Baek YS, Choi JI, Kim YG, et al. Atrial Substrate Underlies the Recurrence after Catheter Ablation in Patients with Atrial Fibrillation. J Clin Med 2020;9(10):1-13. (Article) (In English). DOI: 10.3390/jcm9103164.

55. Heeger CH, Subin B, Wissner E, et al. Second-generation cryoballoon-based pulmonary vein isolation: Lessons from a five-year follow-up. Int J Cardiol 2020;312:73-80. (In eng). DOI: 10.1016/j.ijcard.2020.03.062.

56. Kis Z, Martirosyan M, Hendriks AA, et al. High Cerebrovascular Thromboembolic Event Rate Long after Unsuccessful Catheter Ablation for Atrial Fibrillation. J Atr Fibrillation 2020;13(3):2294. (Article) (In English). DOI: 10.4022/jafib.2294.

57. Kriatselis C, Unruh T, Kaufmann J, et al. Long-term left atrial remodeling after ablation of persistent atrial fibrillation: 7-year follow-up by cardiovascular magnetic resonance imaging. J Interv Card Electrophysiol 2020;58(1):21-27. (In eng). DOI: 10.1007/s10840-019-00584-1.

58. Romero J, Di Biase L, Mohanty S, et al. Long-Term Outcomes of Left Atrial Appendage Electrical Isolation in Patients With Nonparoxysmal Atrial Fibrillation: A Propensity Score-Matched Analysis. Circ Arrhythm Electrophysiol 2020;13(11):e008390. (In eng). DOI: 10.1161/CIRCEP.120.008390.

59. Sawhney V, Schilling RJ, Providencia R, et al. Cryoablation for persistent and longstanding persistent atrial fibrillation: results from a multicentre European registry. Europace 2020;22(3):375-381. (In eng). DOI: 10.1093/europace/euz313.

60. Sugumar H, Nanayakkara S, Chieng D, et al. Arrhythmia recurrence is more common in females undergoing multiple catheter ablation procedures for persistent atrial fibrillation: Time to close the gender gap. Heart Rhythm 2020;17(5 Pt A):692-698. (In eng). DOI: 10.1016/j.hrthm.2019.12.013.

61. Ding WY, Yang PS, Jang E, et al. Impact of abdominal obesity on outcomes of catheter ablation in Korean patients with atrial fibrillation. Int J Clin Pract 2021;75(10):e14696. (In eng). DOI: 10.1111/ijcp.14696.

62. Esato M, An Y, Ogawa H, et al. Major adverse cardiovascular events and mortality after catheter ablation in Japanese patients with atrial fibrillation: The Fushimi AF Registry. Heart and vessels 2021;36(8):1219-1227. (In eng). DOI: 10.1007/s00380-021-01796-0.

63. Gallagher MM, Yi G, Gonna H, et al. Multi-catheter cryotherapy compared with radiofrequency ablation in long-standing persistent atrial fibrillation: a randomized clinical trial. Europace 2021;23(3):370-379. (In eng). DOI: 10.1093/europace/euaa289.

64. Inamura Y, Nitta J, Inaba O, et al. Presence of non-pulmonary vein foci in patients with atrial fibrillation undergoing standard ablation of pulmonary vein isolation: Clinical characteristics and long-term ablation outcome. Int J Cardiol Heart Vasc 2021;32:100717. (In eng). DOI: 10.1016/j.ijcha.2021.100717.

65. Jastrzebski M, Kielbasa G, Fijorek K, et al. Outcomes of atrial fibrillation ablation program based on single-shot techniques. Postepy Kardiol Interwencyjnej 2021;16(4):466-473. (Article) (In English). DOI: 10.5114/aic.2020.101773.

66. Maier J, Blessberger H, Nahler A, et al. Cardiac Computed Tomography-Derived Left Atrial Volume Index as a Predictor of Long-Term Success of Cryo-Ablation in Patients With Atrial Fibrillation. Am J Cardiol 2021;140:69-77. (In eng). DOI: 10.1016/j.amjcard.2020.10.061.

67. Mugnai G, Paparella G, Overeinder I, et al. Long-term clinical outcomes after single freeze cryoballoon ablation for paroxysmal atrial fibrillation: a 5-year follow-up. J Interv Card Electrophysiol 2021;61(1):87-93. (In eng). DOI: 10.1007/s10840-020-00788-w.

68. Šinkovec M, Jan M, Antolič B, Klemen L, Pernat A. Long-term outcomes after catheter ablation of atrial fibrillation: single centre experience. Slovenian Medical Journal 2021;90(1-2):21-37. (Article) (In Slovenian). DOI: 10.6016/ZdravVestn.3148.

69. Wen S, Pislaru C, Monahan KH, et al. Arrhythmia Recurrence After Atrial Fibrillation Ablation: Impact of Warfarin vs. Non-Vitamin K Antagonist Oral Anticoagulants. Cardiovasc Drugs Ther 2021 (Article in Press) (In English). DOI: 10.1007/s10557-021-07200-3.

70. Wu G, Huang H, Cai L, et al. Long-term observation of catheter ablation vs. pharmacotherapy in the management of persistent and long-standing persistent atrial fibrillation (CAPA study). Europace 2021;23(5):731-739. (In eng). DOI: 10.1093/europace/euaa356.

71. Baimbetov AK, Abzaliev KB, Jukenova AM, Bizhanov KA, Bairamov BA, Ualiyeva AY. The efficacy and safety of cryoballoon catheter ablation in patients with paroxysmal atrial fibrillation. Ir J Med Sci 2022;191(1):187-193. (Article in Press) (In English). DOI: 10.1007/s11845-021-02560-z.

72. Schlogl S, Schlogl KS, Haarmann H, et al. Remote magnetic navigation versus manual catheter ablation of atrial fibrillation: A single center long-term comparison. Pacing Clin Electrophysiol 2022;45(1):14-22. (Article in Press) (In English). DOI: 10.1111/pace.14392.

73. Simon J, El Mahdiui M, Smit JM, et al. Left atrial appendage size is a marker of atrial fibrillation recurrence after radiofrequency catheter ablation in patients with persistent atrial fibrillation. Clinical cardiology 2022;45(3):273-281. (Article in Press) (In English). DOI: 10.1002/clc.23748.
